# Rain-Induced Vibration Energy Harvesting Using Nonlinear Plates with Piezoelectric Integration and Power Management

**DOI:** 10.3390/s25144347

**Published:** 2025-07-11

**Authors:** Yi-Ren Wang, Wei Ting Lin, Bo-Jang Huang

**Affiliations:** Department of Aerospace Engineering, Tamkang University, Tamsui District, NewTaipei City 25137, Taiwan; 613430049@o365.tku.edu.tw (W.T.L.); 612430347@o365.tku.edu.tw (B.-J.H.)

**Keywords:** rain energy harvesting, renewable energy, PZT sensors, power management circuit, slapping force

## Abstract

Vibration energy offers promising potential for renewable energy harvesting, especially in conditions where conventional sources such as solar power may be limited or intermittent. This study proposes a rain energy harvester (REH) that converts the kinetic energy of raindrops into electrical energy using nonlinear thin plates, integrated with piezoelectric elements. Two plate configurations—fully hinged (H-H-H-H) and clamped–hinged–free–hinged (C-H-F-H)—are investigated. Theoretical modeling and simulation results are compared with experimental data, with special attention paid to the role of slapping forces in improving prediction accuracy. A power management system is also introduced to stabilize and regulate the harvested voltage. Results confirm the feasibility of rain-induced energy harvesting, showing potential for application in rain-prone areas and integration with existing infrastructure such as solar panels, tents, or canopies.

## 1. Introduction

Vibration phenomena are ubiquitous in nature and are not limited to mechanical components; they can be observed in everyday structures such as bridges, roads, and buildings. As a result, vibration energy is considered a promising form of next-generation green energy. Among green energy solutions, solar panels are widely manufactured and used to collect solar energy. However, in rainy countries, the efficiency of solar panels decreases significantly. If a vibration energy harvesting system could be used to capture the energy from raindrops or be integrated with solar panels, it could not only improve the power generation efficiency of solar panels on rainy days but also enhance their applicability in rain-prone regions. For countries with abundant rainfall, the concept of utilizing raindrop impact power generation can be applied not only to solar panels but also to various structures such as rain shelters, canopies, tents, and convertible car roofs. Increasingly frequent extreme weather has made research on Rain Energy Harvesting Systems (REHs) highly practical. Additionally, using piezoelectric materials to collect vibration energy generated by rainfall has been proven to be a promising and viable approach, leading to the development of vibration energy harvesting systems (VEHs) as a practical application method. This study considers two types of nonlinear plate vibrations: one is a thin plate with all four edges hinged (hinged–hinged–hinged–hinged, H-H-H-H plate), and the other is a thin plate with one clamped edge, two hinged edges, and a free boundary on the opposite side of the fixed edge (clamped–hinged–free–hinged, C-H-F-H plate). These plates are used to capture the kinetic energy of raindrops, with multiple piezoelectric patches placed beneath the vibrating plate to achieve power generation. A nonlinear plate model is derived through theoretical analysis and numerically simulated with a function representing the random impact forces of raindrops. Commercial software simulations are also conducted to determine the best placement of multiple piezoelectric patches. Finally, the power generation efficiency and feasibility of the system are validated through a combination of software simulations and experimental verification. Additionally, this study incorporates a power management (PM) system that selectively outputs the most efficient energy generated from each vibration cycle, ensuring energy storage while enhancing power generation efficiency and reducing unnecessary energy loss. The proposed system can be applied to vibration energy harvesting in solar panels or rain shelters.

Yildirim et al. [1] pointed out that the problem with traditional vibration energy harvesters (VEHs) is that they only operate effectively near resonance, which limits the effective working range for power collection and constrains the total extractable power. This is dependent on the design of the device as well as the amplitude and frequency of the source vibrations. Even if the environmental vibration sources are well-documented and their frequencies are known, the precise manufacturing of the devices is required since small deviations can lead to significant power reductions. Jia [2] suggested that nonlinear vibration energy harvesting systems have a wider effective operating bandwidth and can achieve greater amplitude responses compared to traditional linear systems. Yang and Towfighian [3] demonstrated that nonlinear vibration energy harvesters outperform linear resonators, which perform poorly when operating away from their natural frequencies. Most vibration energy harvesters are linear resonators with narrow bandwidths, leading to significant output declines when the excitation frequency differs from the resonant frequency. Yao et al. [4] reiterated the advantages of nonlinear vibration energy harvesting systems and concluded that nonlinear system designs are essential for improving output power and broadening the frequency response range. Wu et al. [5] summarized the requirements for new energy solutions and the piezoelectric mechanism. In addition to summarizing methods and mechanisms for enhancing piezoelectric power output, they explained the issue of most proposed piezoelectric vibration energy harvesting systems generating only small amounts of electricity. Therefore, with the aim of capturing more vibration energy, this study will focus on nonlinear vibration energy harvesting systems as its primary research direction.

Cao et al. [6] proposed a hybrid wind and rainwater energy harvester (WEREH) system that maintains self-powered operation under various weather conditions. It was installed on the drainage pipes of piers on a cross-sea bridge. On one hand, sensors for health monitoring require substantial power; on the other hand, harsh working environments reduce battery lifespan. Bao et al. [7] pointed out that many designs focused on harvesting energy from high-energy-density sources like mechanical vibrations are not always available for certain application scenarios. The total output power of energy harvesting methods may also be constrained by size limitations. Thus, when such energy sources are unavailable or insufficient for practical applications, alternative energy solutions must be found. Low-energy-density sources such as wind or rain are generally available over large areas and can effectively power small, remote, wireless electronic devices and self-powered sensors. Ong et al. [8] designed a mechanism to guide water droplets into a syringe, thereby converting random raindrops into single droplets that optimally impact the piezoelectric beam. This strategy enhances energy harvesting efficiency by optimizing droplet impact positions. Chilabi et al. [9] analyzed rotational piezoelectric energy harvesting (RPZTEH) and categorized it into four types based on external forces: fluid (air and water) motion, human motion, rotating vehicle tires, and other rotational operational principles. In addition to direct impact, using water as a mechanical input source for RPZTEH can involve an indirect method where PZT turbines or watermills are excited by magnetic forces. Wong et al. [10] found in practical field tests that raindrop impacts form a thin water layer on the surface of piezoelectric beams. This water layer acts as artificial mass and provides resistance to the beam structure while also affecting its dynamic characteristics, such as damping coefficient and vibration mode. The study concluded that energy generation from raindrop impact on piezoelectric harvesters is highly dependent on rainfall rate. Due to the inconsistency of energy production, piezoelectric energy harvesters need to integrate suitable energy storage devices to maintain continuous operation.

Rainfall amount, in addition to rainfall rate, significantly influences the effectiveness of rainwater energy harvesting (REH). Hadidi [11] explored three innovative REH mechanisms: (1) a piezoelectric panel collector converting raindrop kinetic energy into electricity; (2) a tower (similar to a factory chimney) amplifying collected rainwater’s potential energy for turbine-based electricity generation; and (3) a salinity gradient power generation device utilizing the difference between rainwater and saltwater. Ilyas and Swingler [12] categorized the impact mechanisms of REH droplets on solid surfaces into three types: bouncing (complete or partial, leaving varying amounts of water residue), spreading (water adhering to the surface), and splashing (droplet fragmentation and distribution). Recognizing the limitations of single-droplet studies, Wong et al. [13] conducted experiments simulating three rainfall patterns with multiple droplets impacting a piezoelectric beam at various locations. This approach aimed to more realistically model raindrop impacts and their effect on energy harvesting. To further enhance realism, numerical simulation using random numbers will be employed to simulate actual raindrop behavior. Miao and Jia [14] mentioned that in regions with high rainfall, harvesting raindrop energy can be an additional alternative solution to enhance the availability of decentralized energy for sensors or small electronic devices (such as single-device-level remote and wireless communication equipment). It can also supplement traditional decentralized renewable energy generation solutions. Examples of potential candidate regions include the remote northern areas of the Amazon in Brazil and the isolated islands of eastern India and Bangladesh, where seasonal rainfall is abundant, and connecting to the national power grid is both challenging and uneconomical. These effects are further amplified by the cumulative superposition of uniformly distributed raindrops on the plate. For distributed raindrop models, the same cumulative edge strain effects were observed, yielding energy densities several orders of magnitude higher than at single direct impact points. Taiwan also experiences an average annual rainfall of 2457 mm, 2.6 times the global average, as noted by Liaw and Chiang [15]. Rainfall distribution across Taiwan varies considerably, decreasing from northeast to southwest. This regional variation is further complicated by Taiwan’s topography, leading to significant differences even within the same rainfall range. This underscores the necessity of rainfall zoning for accurate rainfall potential assessment. El-Hebeary [16] designed plate structures for harvesting vibration energy from two or more vibration modes. The motivation for this research was to develop resonators that respond to variable-frequency base excitation sources. Duvigneau et al. [17] found unusual results in experimental modal analysis from the oil sump of a twin-cylinder diesel engine. Typically, such a thin oil pan base would exhibit vibration behavior similar to a plate. It was observed that in square plates, there can be positive and negative superpositions of natural modes with the same natural frequency. These superpositions were observed under free-free boundary conditions through analytical, numerical, and experimental methods. Aridogan et al. [18] suggested that compared to harvesters based on piezoelectric cantilever beams, piezoelectric patch harvesters integrated into thin-plate structures could be a more efficient and compact choice for powering wireless structural health and condition monitoring systems. Their study introduced piezoelectric patch-based thin-plate steady-state stochastic vibration energy harvesting, along with electromechanical modeling, analysis, numerical solutions, and experimental validation. They proposed a distributed parameter modeling method for transverse point force-excited thin motherboards, presenting electromechanical models for multiple piezoelectric patches connected in series and parallel.

Wang et al. [19] designed and fabricated a type of film-based converter using polyaniline and polyaniline–graphene composite films to harvest rainwater energy. These converters generated maximum current and voltage outputs of 3.80 μA and 85.01 μV per raindrop, respectively, based on the charging and discharging processes of cations at the rain-to-film interface with the pseudocapacitor. Zhao et al. [20] utilized graphene-rich composite films fabricated through a simple casting technique as self-powered single electrodes to harvest rainwater energy. They successfully developed single-electrode systems that generated periodic current and voltage signals when raindrops impacted the electrodes. Zhu et al. [21] designed a self-powered cathodic protection (CP) system for metal surface protection driven by a triboelectric nanogenerator (TENG), which harvested energy from natural raindrops and wind. The corrosion mechanism on metal surfaces is an electrochemical process that accelerates in the presence of a dilute electrolyte, such as during rainfall. Ahmad et al. [22] suggested that in piezoelectric patch designs, mechanical damping dissipates system energy and weakens vibrations. Reducing mechanical damping can thus improve output power. They found that stiffer piezoelectric patches (with higher elastic modulus) resulted in smaller deflections, leading to reduced output power. Viola [23] conducted experimental comparisons among different rain energy harvesting structures and studied their rectification circuits. Three configurations were considered: cantilever (fixed at one side), bridge (fixed at both ends), and floating ring (a cantilever loop subject to external loading). Edla et al. [24] proposed an improved H-bridge rectification circuit to enhance and stabilize output voltage. To evaluate the circuit’s effectiveness for human-motion energy harvesting, they conducted tests with a piezoelectric device (PD) connected to a cantilever beam and subjected it to low-frequency mechanical vibrations using a shaker. Lu and Boussaid [25] introduced an efficient P-SSHI rectifier for piezoelectric energy harvesting. The rectifier detected the polarity change in current generated by the PD using the voltage across the PD terminals. The voltage reversal process was automatically controlled by diodes in an oscillating network. This achievement provided inspiration for integrating a power management system into rain energy harvesting (REH) systems.

The research regarding energy harvesting from other natural sources of energy is studied by Hollm et al. [26]. They analyze a WEC design with inclined modules connected to a floating frame. Using numerical models, it examines energy harvesting performance under regular and irregular waves, highlighting the effects of design and wave parameters. Ambrożkiewicz et al. [27] use piezoelectric energy harvesting systems to convert mechanical energy into electricity, analyzing factors such as vehicle speed and embedment depth that influence power output in pavement applications. Páez-Montoro et al. [28] present a solar energy harvesting system using a semi-flexible solar module, flexible battery, and BLE microcontroller. Tested under daily use conditions, the device achieved up to 53% charge gain, and could operate continuously with 6 h of direct sunlight per day. Regarding intentional nonlinearities in energy harvesting, Rosso et al. [29] experimentally investigated a magnetically frequency-upconverted piezoelectric energy harvester. Using a low-frequency shaker (3 Hz) (APS 113 Long-Stroke shaker (SPEKTRA Schwingungstechnik und Akustik GmbH Dresden, Germany)), they explored how the velocity of magnetic interaction influenced the activation of the first vibration mode, with nonlinear material effects observed as frequency shifts in the output spectrum. Additionally, Daukševičius et al. [30] analyzed magnetic plucking dynamics for frequency up-conversion, identifying key parameters for efficient energy transfer and demonstrating that transient resonance can maximize power output. These findings provide design insights for wearable energy harvesting applications.

The use of rain-induced excitation is motivated by practical considerations for energy harvesting in environments where solar energy is unreliable due to frequent rainfall—such as in tropical, subtropical, or high-humidity regions. While other types of excitation (e.g., wind, vibration from human activity, or mechanical shaking) are widely studied, rainfall is an abundant and underutilized energy source that naturally produces localized, impulsive forces suitable for thin-plate vibration. Moreover, raindrops generate non-periodic, stochastic impacts, which makes this type of loading particularly challenging and novel for modeling. By successfully harvesting energy under such irregular excitation, this study demonstrates a potentially valuable alternative energy source for low-power devices (e.g., environmental sensors, IoT systems) in outdoor, rain-prone environments. The ability to integrate this system with existing structures (such as tents, canopies, or solar panels) further supports its practical appeal. This present study first establishes a theoretical plate motion model to verify the feasibility of the proposed “Rain Energy Harvester (REH)” system through numerical calculations, commercial software simulations, and experimental validation. Given that the plate thickness is much smaller than its length and width, the Kirchhoff–Love Plate Theory was selected as the primary framework. Random functions simulating raindrops were incorporated into the numerical simulations to develop a model that describes plate deformation caused by the kinetic energy of raindrops. This study analyzes two plate configurations: a fully hinged plate (H-H-H-H plate) and a plate with one fixed edge, two hinged edges, and one free edge (C-H-F-H plate) to simulate the vibration behavior of solar panels and/or rain covers. Using ANSYS Workbench (ANSYS 2021 R1) for simulation, the study identifies the best piezoelectric patch placements and evaluates their piezoelectric outputs. Numerical simulations incorporating piezoelectric equations further validate these findings. In the experimental setup, elastic steel was used as the plate material. Multiple small steel balls were dropped at various locations on the plate to simulate random raindrop impacts. A power management (PM) system was integrated, involving a full-bridge rectifier to convert the alternating current from the piezoelectric patches into direct current. The rectified current was filtered using an Arduino Uno R3 (Mouser electronics, Inc., Taipei, Taiwan), which selected the highest energy-generating vibration events. These signals were then fed into a buck-boost converter to provide a stable voltage for circuit loads or battery storage. The PM system ensures efficient vibration energy capture, stable conversion, and storage, providing a reliable voltage output. This study conducts mutual validation through numerical calculations, software simulations, and experimental results. This research will offer practical applications for rain-rich regions. Potential applications include integrating the system with solar panels to enhance energy efficiency on rainy days or using it for power generation in structures like tents and rain shelters. The system is also suitable for remote areas without access to the power grid or emergency power supply scenarios after disasters, providing decentralized and sustainable energy solutions. Moreover, this study can drive industrial applications in piezoelectric materials and energy management technologies, fostering the design and production of green energy equipment and stimulating related industrial developments. For smart sensor and IoT applications, a miniaturized rain energy harvesting system can power small sensors and IoT devices, such as those for bridge health monitoring, agricultural sensing, and environmental monitoring, enhancing the devices’ autonomy and practicality. Given the study’s methodological and theoretical extensibility, the proposed approach can be adapted to other vibration energy harvesting scenarios, such as wind energy and structural vibrations, offering foundational models and methodologies for future research in emerging energy technologies.

The remainder of this paper is organized as follows. Section 2 presents the theoretical modeling of the nonlinear plate using Hamilton’s principle, including geometric assumptions, governing equations, and piezoelectric coupling. Section 3 describes the numerical methods used to solve the system, including the application of random excitation forces and the Runge–Kutta algorithm. Section 4 describes the experimental setup, including the vibration energy harvester, piezoelectric patch placement, and measurement procedures. It also compares theoretical, simulation, and experimental results, focusing on the effects of slapping forces and boundary conditions. Section 5 introduces the power management system design, including rectification and NMOS control for voltage stabilization and efficient energy handling. Finally, Section 6 provides conclusions and discusses implications for future development.

## 2. Derivation of Nonlinear Equations

**Figure 1:**
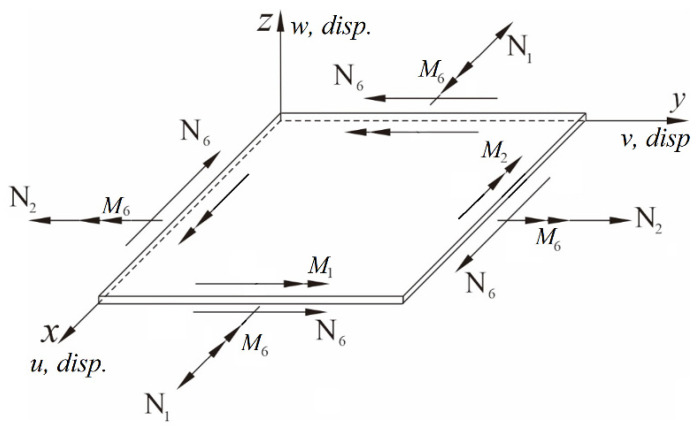
Schematic diagram of plate displacement, internal force, and moment.

Based on Kirchhoff’s Classical Plate Theory, and utilizing the Hamilton principle along with von Kármán-type nonlinear strain terms, the nonlinear plate is taken as the focus. The derivation references the small perturbation theory for nonlinear plates proposed by Nayfeh and Pai [31] and Du [32]. Assuming that the plate is made of a homogeneous material and that its thickness is much smaller than its length and width, planar motion is assumed for the plate. The displacements along the three axes (*x*, *y*, *z*) are denoted as *u*, *v*, and *w*, respectively. Internal forces are represented by *N*_1_, *N*_2_ and *N*_6_, while moments are denoted by *M*_1_, *M*_2_, and *M*_6_, as shown in Figure 1. The equations for the plate along the three axes can be expressed as follows:

*x*-direction:

(1) $$N_{1x}+N_{6y}=I_{0}\ddot{u}-I_{1}{\ddot{w}}_{x}$$

*y*-direction:

(2) $$N_{6x}+N_{2y}=I_{0}\ddot{v}-I_{1}{\ddot{w}}_{y}$$

*z*-direction:

(3) $$M_{1xx}+2M_{6xy}+M_{2yy}+{\left(N_{1}w_{x}+N_{6}w_{y}\right)}_{x}+{\left(N_{6}w_{x}+N_{2}w_{y}\right)}_{y}=I_{0}\ddot{w}-I_{2}({\ddot{w}}_{xx}+{\ddot{w}}_{yy})+I_{1}{\ddot{u}}_{x}+I_{1}{\ddot{v}}_{y}$$

where the subscripts *x* and *y* represent partial differentiation with respect to *x* and *y*, *respectively*. Additionally, *I* can be expressed as follows:

(4) $$\{I_{0},I_{1},I_{2}\}=\int_{z}\rho \{1,z,z^{2}\}dz$$

According to the assumptions of Kirchhoff’s classical plate theory, *I*_1_ = *I*_2_ = 0. Since the plate is isotropic, the internal forces and moments can be expressed as follows:

(5) $$\left\{\begin{array}{l} N_{1}\\ N_{2}\\ N_{6} \end{array}\right\}=\frac{Eh}{1-\nu^{2}}\left[\begin{array}{ccc} 1 & \nu & 0\\ \nu & 1 & 0\\ 0 & 0 & (1-\nu )/2 \end{array}\right]\left\{\begin{array}{l} u_{x}+\frac{1}{2}w_{x}^{2}\\ v_{y}+\frac{1}{2}w_{y}^{2}\\ u_{y}+v_{x}+w_{x}w_{y} \end{array}\right\}$$

(6) $$\left\{\begin{array}{l} M_{1}\\ M_{2}\\ M_{6} \end{array}\right\}=\frac{Eh^{3}}{12(1-\nu^{2})}\left[\begin{array}{ccc} 1 & \nu & 0\\ \nu & 1 & 0\\ 0 & 0 & (1-\nu )/2 \end{array}\right]\left\{\begin{array}{l} w_{xx}\\ w_{yy}\\ 2w_{xy} \end{array}\right\}$$

### 2.1. Theoretical Background of Nonlinear Plate Modeling

This section provides the theoretical foundation necessary for the subsequent development of the nonlinear vibration model and its application to different plate configurations analyzed in this study. Substituting Equations (4)–(6) into Equations (1)–(3) yields the following expressions:

*x*-direction:

(7) $$\frac{\partial^{2}u}{\partial x^{2}}+\frac{1}{2}\left(1+\nu \right)\frac{\partial^{2}v}{\partial x\partial y}+\frac{1}{2}\left(1-\nu \right)\frac{\partial^{2}u}{\partial y^{2}}=-\frac{\partial w}{\partial x}\frac{\partial^{2}w}{\partial x^{2}}-\frac{1}{2}\left(1+\nu \right)\frac{\partial w}{\partial y}\frac{\partial^{2}w}{\partial x\partial y}-\frac{1}{2}\left(1-\nu \right)\frac{\partial w}{\partial x}\frac{\partial^{2}w}{\partial y^{2}}$$

*y*-direction:

(8) $$\frac{\partial^{2}v}{\partial y^{2}}+\frac{1}{2}\left(1+\nu \right)\frac{\partial^{2}u}{\partial x\partial y}+\frac{1}{2}\left(1-\nu \right)\frac{\partial^{2}v}{\partial x^{2}}=-\frac{\partial w}{\partial y}\frac{\partial^{2}w}{\partial y^{2}}-\frac{1}{2}\left(1+\nu \right)\frac{\partial w}{\partial x}\frac{\partial^{2}w}{\partial x\partial y}-\frac{1}{2}\left(1-\nu \right)\frac{\partial w}{\partial y}\frac{\partial^{2}w}{\partial x^{2}}$$

*z*-direction:

(9) $$\begin{array}{l} D\left(\frac{\partial^{4}w}{\partial x^{4}}+2\frac{\partial^{4}w}{\partial x^{2}\partial y^{2}}+\frac{\partial^{4}w}{\partial y^{4}}\right)=F-\rho h\frac{\partial^{2}w}{\partial t^{2}}-\varsigma \frac{\partial w}{\partial t}+\frac{Eh}{1-\nu^{2}}\left\{\left(\frac{\partial u}{\partial x}+\frac{1}{2}{\frac{\partial w}{\partial x}}^{2}\right)\frac{\partial^{2}w}{\partial x^{2}}+\nu \left(\frac{\partial v}{\partial y}+\frac{1}{2}{\frac{\partial w}{\partial y}}^{2}\right)\frac{\partial^{2}w}{\partial x^{2}}\right.\\ \left(\frac{\partial v}{\partial y}+\frac{1}{2}{\frac{\partial w}{\partial y}}^{2}\right)\frac{\partial^{2}w}{\partial y^{2}}+\nu \left(\frac{\partial u}{\partial x}+\frac{1}{2}{\frac{\partial w}{\partial x}}^{2}\right)\frac{\partial^{2}w}{\partial y^{2}}\left.\left(1-\nu \right)\left(\frac{\partial u}{\partial y}+\frac{\partial v}{\partial x}+\frac{\partial w}{\partial x}\frac{\partial w}{\partial y}\right)\frac{\partial^{2}w}{\partial x\partial y}\right\} \end{array}$$

where ρ is the material density of the plate, *h* is the plate thickness, *F* represents the force due to rainwater kinetic energy, ς is the structural damping, and *D* is the plate bending stiffness, expressed as $\frac{Eh^{3}}{12\left(1-\nu^{2}\right)}$, where *E* is the Young’s modulus, and ν is Poisson’s ratio. To facilitate analysis, Equation (9) is non-dimensionalized. Let the dimensionless parameters be α=x/a, β=y/b ,τ=ωt, while retaining the displacement notation *w* from the original equation. The material density, plate thickness, rainwater kinetic energy force, structural damping, and Young’s modulus are non-dimensionalized while preserving their original symbols. Dividing the equation by the dimensionless value *D* of the plate and considering the relationship between length and width where *b* *a* = *b*, the equation width *b* is uniformly expressed as *a*. After simplification, the equation becomes:

(10) $$\begin{array}{l} \left(\frac{\partial^{4}w}{\partial \alpha^{4}}+2\frac{\partial^{4}w}{\partial \alpha^{2}\partial \beta^{2}}+\frac{\partial^{4}w}{\partial \beta^{4}}\right)=\frac{F}{Da^{4}}-\frac{\rho h}{Da^{4}}\frac{\partial^{2}w}{\partial \tau^{2}}-\frac{\varsigma }{Da^{4}}\frac{\partial w}{\partial \tau }+\frac{1}{ah^{2}}\left\{12\frac{\partial u}{\partial \alpha }\frac{\partial^{2}w}{\partial \alpha^{2}}+6a{\left(\frac{\partial w}{\partial \alpha }\right)}^{2}\frac{\partial w}{\partial \alpha^{2}}\right.\\ +12\nu \left(\frac{\partial v}{\partial \beta }\frac{\partial w}{\partial \alpha^{2}}+\frac{1}{2}a{\left(\frac{\partial w}{\partial \beta }\right)}^{2}\frac{\partial^{2}w}{\partial \alpha^{2}}\right)+12\frac{\partial v}{\partial \beta }\frac{\partial^{2}w}{\partial \beta^{2}}+6a{\left(\frac{\partial w}{\partial \beta }\right)}^{2}\frac{\partial w}{\partial \beta^{2}}+12\nu \left(\frac{\partial u}{\partial \alpha }\frac{\partial w}{\partial \beta^{2}}+\frac{1}{2}a{\left(\frac{\partial w}{\partial \alpha }\right)}^{2}\frac{\partial^{2}w}{\partial \beta^{2}}\right)\\ \left.+12(1-\nu )\left(\frac{\partial u}{\partial \beta }\frac{\partial^{2}w}{\partial \alpha \beta }+\frac{\partial v}{\partial \alpha }\frac{\partial^{2}w}{\partial \alpha \beta }+a\frac{\partial w}{\partial \alpha }\frac{\partial w}{\partial \beta }\frac{\partial^{2}w}{\partial \alpha \beta }\right)\right\} \end{array}$$

Using simplified notation, Equation (10) becomes

(11) $$\begin{array}{l} w_{\alpha \alpha \alpha \alpha }+2w_{\alpha \alpha \beta \beta }+w_{\beta \beta \beta \beta }=\tilde{F}-\gamma \ddot{w}-\zeta \dot{w}+\varepsilon \left\{12u_{\alpha }w_{\alpha \alpha }+6aw_{\alpha }^{2}w_{\alpha \alpha }+12\nu \left(v_{\beta }w_{\alpha \alpha }+\frac{1}{2}aw_{\beta }^{2}w_{\alpha \alpha }\right)\right.\\ +12v_{\beta }w_{\beta \beta }+6aw_{\beta }^{2}w_{\beta \beta }+12\nu \left(u_{\alpha }w_{\beta \beta }+a\frac{1}{2}w_{\alpha }^{2}w_{\beta \beta }\right)\left.+12(1-\nu )\left(u_{\beta }w_{\alpha \beta }+v_{\alpha }w_{\alpha \beta }+aw_{\alpha }w_{\beta }w_{\alpha \beta }\right)\right\} \end{array}$$

(12) $$\tilde{F}=\frac{F}{Da^{4}},\;\gamma =\frac{\rho h}{Da^{4}},\;\zeta =\frac{\varsigma }{Da^{4}},\;\varepsilon =\frac{h^{2}}{a^{2}}$$

For the plate, ζ represents the structural damping coefficient. Setting *a* = *b* = 1 (dimensionless), Equation (11) can be further simplified as follows:

(13) $$\begin{array}{l} w_{\alpha \alpha \alpha \alpha }+2w_{\alpha \alpha \beta \beta }+w_{\beta \beta \beta \beta }=\tilde{F}-\gamma \ddot{w}-\zeta \dot{w}+\varepsilon \left\{12u_{\alpha }w_{\alpha \alpha }+6w_{\alpha }^{2}w_{\alpha \alpha }+12\nu \left(v_{\beta }w_{\alpha \alpha }+\frac{1}{2}w_{\beta }^{2}w_{\alpha \alpha }\right)\right.\\ +12v_{\beta }w_{\beta \beta }+6w_{\beta }^{2}w_{\beta \beta }+12\nu \left(u_{\alpha }w_{\beta \beta }+\frac{1}{2}w_{\alpha }^{2}w_{\beta \beta }\right)\left.+12(1-\nu )\left(u_{\beta }w_{\alpha \beta }+v_{\alpha }w_{\alpha \beta }+w_{\alpha }w_{\beta }w_{\alpha \beta }\right)\right\} \end{array}$$

A. Nonlinear equation for H-H-H-H plate

Considering a square simply supported plate (H-H-H-H plate) with side lengths *a* and *b* where *a* = *b*, the boundary conditions are as follows:

(14) $$w\left(x,0,t\right)=w\left(x,b,t\right)$$

(15) $$w\left(0,y,t\right)=w\left(a,y,t\right)$$

(16) $$\frac{\partial^{2}w}{\partial x^{2}}(0,y,t)=\nu \frac{\partial^{2}w}{\partial y^{2}}(a,y,t)$$

(17) $$\frac{\partial^{2}w}{\partial x^{2}}(x,0,t)=\nu \frac{\partial^{2}w}{\partial y^{2}}(x,b,t)$$

Using the boundary conditions from Equations (14)–(17), to ensure that Equation (13) only contains a representation of *w*, it is necessary to first derive the equations of motion for *u* and *v*. This involves assuming the vibration mode of the plate as follows:

(18) w=η(τ)sinmπαsinnπβ+ξ(τ)sinnπαsinmπβ

In which, η(τ) and ξ(τ) are the time-dependent modal functions, and α, β are the dimensionless spatial coordinates. Equation (18) represents the assumed mode shape for the H-H-H-H plate. By substituting this mode shape into the nondimensional forms of Equations (7) and (8), the deformation equations in the *u*- and *v*-directions can be derived. Subsequently, substituting the expressions for *u* and *v* into Equation (13) yields the nondimensional nonlinear plate motion equation, which can be written as follows:

(19) $$\begin{array}{l} w_{\alpha \alpha \alpha \alpha }+2w_{\alpha \alpha \beta \beta }+w_{\beta \beta \beta \beta }=\tilde{F}-\gamma \ddot{w}-\zeta \dot{w}+\varepsilon \left\{12u_{\alpha }w_{\alpha \alpha }+6w_{\alpha }^{2}w_{\alpha \alpha }+12\nu \left(v_{\beta }w_{\alpha \alpha }+\frac{1}{2}w_{\beta }^{2}w_{\alpha \alpha }\right)\right.\\ +12v_{\beta }w_{\beta \beta }+6w_{\beta }^{2}w_{\beta \beta }+12\nu \left(u_{\alpha }w_{\beta \beta }+\frac{1}{2}w_{\alpha }^{2}w_{\beta \beta }\right)\left.+12(1-\nu )\left(u_{\beta }w_{\alpha \beta }+v_{\alpha }w_{\alpha \beta }+w_{\alpha }w_{\beta }w_{\alpha \beta }\right)\right\} \end{array}$$

For details, please refer to Appendix A.

B. Nonlinear C-H-F-H plate equation

Consider a square clamped–hinged–free–hinged plate (C-H-F-H plate) with dimensions *a* and *b* where *a* = *b*. The displacement equations for *u*, *v* and *w* can be assumed to be $u=\bar{u}\phi_{n}(x)\phi_{m}(y)$, $v=\bar{v}\phi_{m}(y)\phi_{n}(x)$, $w=A(t)\phi_{n}(x)\phi_{m}(y)$, where A(t) represents the amplitude in the *w*-direction, and $\bar{u}$ and $\bar{v}$ are the vibration amplitudes coupled in the *x*-direction and *y*-direction of the plate. Here, $\phi_{n}(x)$ represents the vibration mode shape in the *x*-direction, with boundary conditions of clamped–free as shown in Equation (20). Similarly, $\phi_{m}(y)$ represents the vibration mode shape in the *y*-direction, with Hinged-Hinged boundary conditions as shown in Equation (21).

(20) $$\phi_{n}(x)=\sin E_{n}x-\sinh E_{n}x-H_{n}(\cos E_{n}x-\cosh E_{n}x),\;H_{n}=\frac{\left(\sin E_{n}l+\sinh E_{n}l\right)}{\left(\cos E_{n}l+\cosh E_{n}l\right)}$$

(21) $$\phi_{m}(y)=\sin(m\pi y)$$

Let α=x/a, β=y/b, τ=ωt, and continue using the original displacement notation *w*. Next, by substituting $u=\bar{u}\phi_{n}(\alpha )\phi_{m}(\beta )$ and $v=\bar{v}\phi_{m}(\beta )\phi_{n}(\alpha )$ into Equations (7) and (8), and following the same procedure as for the H-H-H-H plate, we obtain the expressions for *u*, *v*, and $w=A(t)\phi_{n}(\alpha )\phi_{m}(\beta )$. After nondimensionalization, these expressions are then substituted into the nonlinear plate motion equation (Equation (13)), with the addition of a damping term, where *μ* denotes the damping coefficient. This yields the nonlinear plate equation with boundary conditions specific to the clamped–hinged–free–hinged (C-H-F-H) plate. The resulting amplitude function (*A*(*t*)) and spatial mode $\phi_{n}(\alpha )\phi_{m}(\beta )$, after discretization, are expressed as follows:

(22) $$\ddot{A}Q_{1}(\alpha ,\beta )+\dot{A}Q_{2}(\alpha ,\beta )+AQ_{3}(\alpha ,\beta )-A^{3}Q_{4}(\alpha ,\beta )=\tilde{F}(t)$$

For details, please refer to Appendix B. Here, *A* represents the amplitude in the *w*-direction, $\tilde{F}(t)$ represents the external force from the raindrops, and $Q_{1}(\alpha ,\beta )∼Q_{4}(\alpha ,\beta )$ are defined in Appendix C.

### 2.2. Analysis of Raindrop External Force Disturbance

The external force in this study originates from the impact force *F*(*t*) of falling raindrops, which is expressed as follows:

(23) $$F(t)={\left\{F_{weight}\cdot R(i,j)\;\right\}}_{t}\;t=1,2,\ldots ,10$$

In Equation (23), *F*(*t*) represents the external force acting on the plate at time *t*, and it is derived from randomly selected values *R*(*i*, *j*) from a 100 × 100 matrix. The {}_*t*_ represents varying external force disturbances per unit time. There is no summation over *i* and *j* in this formulation. Rather, at each time step *t*, a single localized force is applied to the plate using one randomly chosen (*i*, *j*) position from the matrix. Therefore, the use of *R*(*i*, *j*) reflects a point-wise impact force rather than a spatially integrated one. The matrix *R*(*i*, *j*) numerically represents the spatial discretization of the plate surface into 100 × 100 positions. Each element of this matrix is generated using the additive congruential method to produce uniformly distributed pseudo-random values between 0 and 1. These values are used to simulate the random spatial and temporal distribution of raindrop impacts on the plate surface. At each time step, one random location (*i*, *j*) is selected, and the corresponding *R*(*i*, *j*) value is used to define the magnitude of the applied force, simulating a localized raindrop impact on the plate surface. In our simulations, the model considers 10 discrete raindrop impacts over time (i.e., *t* = 1 to 10) per set, which represents a short-duration random excitation event. To enhance statistical reliability, multiple sets of independent simulations were carried out, totaling several tens of simulation runs. While the duration is limited in this study, the trends align well with experimental data. The approach serves as a proof-of-concept for modeling random rain excitation. Assuming the raindrop consists of pure water and retains a spherical shape under standard atmospheric pressure and room temperature conditions. The gravitational force *F_weight_* of the raindrop is defined as follows:

(24) $$F_{weight}=mg=\rho_{water}\frac{4\pi r^{3}}{3}g$$

where *m* is the mass of the raindrop, *g* is the gravitational constant, *r* is the radius of the raindrop, and $\rho_{water}$ is the density of water (1000 kg/m^3^). When raindrops fall, they are affected by various external factors such as air resistance and crosswinds. Additionally, the size of raindrops is difficult to measure accurately. Therefore, the impact force of falling raindrops is considered unpredictable. To describe the impact force of raindrops, this study uses random numbers for simulation. The additive congruential method is employed to generate pseudo-random numbers, expressed as follows:

(25) $$R_{n+1}=(2^{\upsilon }-1)R_{n}+\frac{c[{\left(2^{\upsilon }+1\right)}^{n}-1]}{2^{\upsilon }}\;{(\mathrm{mod}\;2}^{P})$$

where *R_n+_*_1_ is the (*n* + 1)-th generated random number, *R_n_* is the current random number, υ is a constant and controls the growth rate of the sequence, *c* ensures the maximum period of the random number sequence (typically an odd number), *P* is the bit length of binary numbers in the computer (excluding the sign bit). To describe the impact force of raindrops, this study uses random numbers simulated via the additive congruential method [33], a classic algorithm in pseudo-random number generation. The specific form used is shown in Equation (25), derived following the formulation in [33]. In this study, a pseudo-random force model is employed to simulate the stochastic nature of raindrop impacts on the plate surface. The random excitation represents variations in drop arrival timing, impact location, and force magnitude. Although falling raindrops are known to reach a terminal velocity due to air resistance, which depends on droplet size and falling height, the current model simplifies these effects into a series of discrete, randomly distributed impulses. This abstraction avoids the need for detailed fluid-dynamic modeling while preserving the essential randomness of real-world rainfall. The use of random forces allows us to evaluate the energy harvesting behavior under non-deterministic excitation.

Using the above pseudo-random number sequence, the computer generates ten sets of 100 × 100 matrices *R*(*i*, *j*), where each element in the matrix is a random number. Each matrix represents time-varying random external force disturbances used in this study.

### 2.3. Theoretical Model Construction of Piezoelectric Patch Equations

This section establishes the piezoelectric equations for the piezoelectric patch. Only the Coulomb force exerted by the piezoelectric patch on the nonlinear cantilever beam is considered. According to the study by Rajora [34], the piezoelectric equation for the piezoelectric patch is given as follows:

(26) $$c_{p}\dot{V}+\frac{1}{{\bar{R}}_{p}}V+\int_{\hat{a}}^{\hat{b}}eh_{p}t_{h}{\dot{\bar{w}}}^{\prime \prime }d\bar{x}=0$$

where *c_p_* is the capacitance of the piezoelectric patch, *V* is the voltage across the load resistor, ${\bar{R}}_{p}$ is the load resistance, *e* is the dielectric constant, *h_p_* is the length of the piezoelectric patch, *t_h_* is the thickness of the piezoelectric patch, $\hat{a}$ and $\hat{b}$ represent the positions of both ends of the piezoelectric patch. In this study, the piezoelectric patch is placed at the root of the plate, meaning $\hat{a}$ = 0 and $\hat{b}$ equals the length of the piezoelectric patch. Next, the Coulomb force exerted by the piezoelectric patch on the plate is discussed. The third term on the left-hand side of Equation (26), representing the Coulomb force exerted by the piezoelectric patch on the plate, can be expressed as follows:

(27) $$\int_{\hat{a}}^{\hat{b}}eh_{p}t_{h}{\dot{\bar{w}}}^{\prime \prime }d\bar{x}=\left(et_{h}\int_{\hat{a}}^{\hat{b}}{\dot{\bar{w}}}^{\prime \prime }d\bar{x}\right)=c_{f}\left(\int_{\hat{a}}^{\hat{b}}{\dot{\bar{w}}}^{\prime \prime }d\bar{x}\right)V$$

In Equation (27), *c_f_* represents the piezoelectric coupling coefficient. Substituting Equation (27) into Equation (26), and then nondimensionalizing the equation using the factor *c_p_ c_f_*ω, yields:

(28) $$\dot{v}+R_{p}v+\hat{k}\int_{\tilde{a}}^{\tilde{b}}{\dot{w}}^{\prime \prime }d\alpha =0$$

where $v=V/c_{f}$ is the dimensionless voltage, ω is the vibration frequency of the plate, $R_{p}=1/{\bar{R}}_{p}c_{p}\omega$ is the dimensionless resistance, $\hat{k}=eh_{p}t_{h}/c_{p}c_{f}$, $(\;\dot{)}=d/d\tau$ represents differentiation with respect to dimensionless time, ()′ represents differentiation with respect to spatial variables. Depending on the placement of the piezoelectric patch, differentiation can be expressed as d/dα or d/dβ, with the relationships $\tilde{a}=\hat{a}/l$ and $\tilde{b}=\hat{b}/l$. After further computation and reorganization, using the same notation for both dimensional and dimensionless forms, Equation (28) is a first-order ordinary differential equation (ODE), which can be analytically solved to obtain:

(29) $$v=-\frac{\hat{k}}{e^{R_{p}\tau }}\int_{0}^{\tau }\left(\int_{\tilde{a}}^{\tilde{b}}{\dot{w}}^{\prime \prime }d\alpha \right)e^{R_{p}\tau }d\tau$$

Substituting Equation (29) into Equation (26) and dividing both sides accordingly by $lm\omega^{2}$, the dimensionless Coulomb force can be obtained. Let $\eta^{2}=\frac{c_{f}^{2}}{lm\omega^{2}}$ and after simplification, we obtain

(30) $$\frac{c_{f}^{2}\left(\int_{\tilde{a}}^{\tilde{b}}w^{\prime \prime }d\alpha \right)v}{lm\omega^{2}}=\eta^{2}\left(\int_{\tilde{a}}^{\tilde{b}}w^{\prime \prime }d\alpha \right)v=\eta^{2}\left(\int_{\tilde{a}}^{\tilde{b}}w^{\prime \prime }d\alpha \right)\left(-\frac{\hat{k}}{e^{R_{p}\tau }}\int_{0}^{\tau }\left(\int_{\tilde{a}}^{\tilde{b}}\dot{w^{\prime \prime }}d\alpha \right)e^{R_{p}\tau }d\tau \right)=-\frac{\hat{k}}{e^{R_{p}\tau }}\int_{\tilde{a}}^{\tilde{b}}w^{\prime \prime }d\alpha \int_{0}^{\tau }\left(\int_{\tilde{a}}^{\tilde{b}}\dot{w^{\prime \prime }}d\alpha \right)e^{R_{p}\tau }d\tau$$

Next, since the plate is equipped with multiple piezoelectric patches, let the length and width of the plate be equal and normalized to a dimensionless coefficient of 1. The relationship between the length of the piezoelectric patch and the length of the plate is set to 1:10.

A. For the H-H-H-H plate

The positions of the piezoelectric patches are placed at the edges and corners as follows:

- Edges: (0, 0.5), (1, 0.5), (0.5, 0), (0.5, 1)
- Corners: (0, 0), (1, 0), (0, 1), (1, 1)

**Figure 2:**
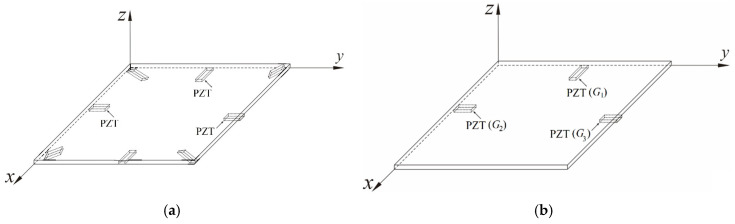
Schematic diagram of piezoelectric patch placement (**a**) on H-H-H-H plate; (**b**) on C-H-F-H plate.

As shown in Figure 2a.

Therefore, Equation (30) corresponding to the positions at the four edges can be written as follows:

(31) $$-\frac{\hat{k}}{e^{R_{p}\tau }}\int_{0.0}^{0.1}{\left.w^{\prime \prime }\right|}_{y=0.5}dx\int_{0}^{\tau }\left(\int_{0.0}^{0.1}{\left.\dot{w^{\prime \prime }}\right|}_{y=0.5}dx\right)e^{R_{p}\tau }d\tau$$

(32) $$-\frac{\hat{k}}{e^{R_{p}\tau }}\int_{0.9}^{1.0}{\left.w^{\prime \prime }\right|}_{y=0.5}dx\int_{0}^{\tau }\left(\int_{0.9}^{1.0}{\left.\dot{w^{\prime \prime }}\right|}_{y=0.5}dx\right)e^{R_{p}\tau }d\tau$$

(33) $$-\frac{\hat{k}}{e^{R_{p}\tau }}\int_{0.0}^{0.1}{\left.w^{\prime \prime }\right|}_{x=0.5}dy\int_{0}^{\tau }\left(\int_{0.0}^{0.1}{\left.\dot{w^{\prime \prime }}\right|}_{x=0.5}dy\right)e^{R_{p}\tau }d\tau$$

(34) $$-\frac{\hat{k}}{e^{R_{p}\tau }}\int_{0.9}^{1.0}{\left.w^{\prime \prime }\right|}_{x=0.5}dy\int_{0}^{\tau }\left(\int_{0.9}^{1.0}{\left.\dot{w^{\prime \prime }}\right|}_{x=0.5}dy\right)e^{R_{p}\tau }d\tau$$

For the four edges can be written as follows:

(35) $$-\frac{\hat{k}}{e^{R_{p}\tau }}\int_{0.0}^{c}\int_{0.0}^{c}w^{\prime \prime }dxdy\int_{0}^{\tau }\left(\int_{0.0}^{c}\int_{0.0}^{c}\dot{w^{\prime \prime }}dxdy\right)e^{R_{p}\tau }d\tau$$

(36) $$-\frac{\hat{k}}{e^{R_{p}\tau }}\int_{0.0}^{c}\int_{d}^{1.0}w^{\prime \prime }dxdy\int_{0}^{\tau }\left(\int_{0.0}^{c}\int_{d}^{1.0}\dot{w^{\prime \prime }}dxdy\right)e^{R_{p}\tau }d\tau$$

(37) $$-\frac{\hat{k}}{e^{R_{p}\tau }}\int_{d}^{1.0}\int_{0.0}^{c}w^{\prime \prime }dxdy\int_{0}^{\tau }\left(\int_{d}^{1.0}\int_{0.0}^{c}\dot{w^{\prime \prime }}dxdy\right)e^{R_{p}\tau }d\tau$$

(38) $$-\frac{\hat{k}}{e^{R_{p}\tau }}\int_{d}^{1.0}\int_{d}^{1.0}w^{\prime \prime }dxdy\int_{0}^{\tau }\left(\int_{d}^{1.0}\int_{d}^{1.0}\dot{w^{\prime \prime }}dxdy\right)e^{R_{p}\tau }d\tau$$

where

(39) $$c=\frac{0.1}{\sqrt{2}},\;d=1-\frac{0.1}{\sqrt{2}}$$

By substituting the piezoelectric patch external force terms from Equations (31)–(38) into the plate’s motion equation (Equation (23)), the resulting non-dimensionalized equation for the nonlinear piezoelectric plate is obtained as follows:

(40) $$\begin{array}{l} w_{\alpha \alpha \alpha \alpha }+2w_{\alpha \alpha \beta \beta }+w_{\beta \beta \beta \beta }=\tilde{F}-\gamma \ddot{w}-\zeta \dot{w}+\varepsilon \left\{12u_{\alpha }w_{\alpha \alpha }+6w_{\alpha }^{2}w_{\alpha \alpha }+12\nu \left(v_{\beta }w_{\alpha \alpha }+\frac{1}{2}w_{\beta }^{2}w_{\alpha \alpha }\right)\right.\\ +12v_{\beta }w_{\beta \beta }+6w_{\beta }^{2}w_{\beta \beta }+2\nu \left(u_{\alpha }w_{\beta \beta }+\frac{1}{2}w_{\alpha }^{2}w_{\beta \beta }\right)\left.+12(1-\nu )\left(u_{\beta }w_{\alpha \beta }+v_{\alpha }w_{\alpha \beta }+w_{\alpha }w_{\beta }w_{\alpha \beta }\right)\right\}+P \end{array}$$

where *P* is the sum of the terms from Equations (31)–(38), which represent the total external force exerted by the piezoelectric patches.

B. For the C-H-F-H plate:

Firstly, the first piezoelectric patch (*G*_1_) is placed on the clamped edge, with α = 0.0 and β = 0.5 (dimensionless position), as shown in Figure 2b. The dimensionless Coulomb force exerted by the piezoelectric patch on the nonlinear plate can be expressed as follows:

(41) $$G_{1}=\eta^{2}(\int_{0.0}^{0.1}{\left.w^{\prime \prime }\right|}_{y=0.5}d\alpha )(-\frac{\hat{k}}{e^{R_{p}\tau }}\int_{0}^{\tau }(\int_{0.0}^{0.1}{\left.{\dot{w}}^{\prime \prime }\right|}_{y=0.5}d\alpha )e^{R_{p}\tau }d\tau )$$

The second piezoelectric patch (*G*_2_) is placed on the hinged edge, with α = 0.5 and β = 0.0. The dimensionless Coulomb force exerted by this piezoelectric patch on the nonlinear plate can be expressed as follows:

(42) $$G_{2}=\eta^{2}(\int_{0.0}^{0.1}{\left.w^{\prime \prime }\right|}_{x=0.5}d\beta )(-\frac{\hat{k}}{e^{R_{p}\tau }}\int_{0}^{\tau }(\int_{0.0}^{0.1}{\left.{\dot{w}}^{\prime \prime }\right|}_{x=0.5}d\beta )e^{R_{p}\tau }d\tau )$$

The third piezoelectric patch (*G*_3_) is placed on the other hinged edge, with α = 0.5 and β = 1. The dimensionless Coulomb force exerted by this piezoelectric patch on the nonlinear plate can be expressed as follows:

(43) $$G_{3}=\eta^{2}(\int_{0.9}^{1.0}{\left.w^{\prime \prime }\right|}_{x=0.5}d\beta )(-\frac{\hat{k}}{e^{R_{p}\tau }}\int_{0}^{\tau }(\int_{0.9}^{1.0}{\left.{\dot{w}}^{\prime \prime }\right|}_{x=0.5}d\beta )e^{R_{p}\tau }d\tau )$$

By directly combining Equations (24)–(28), the piezoelectric coupling equation for the C-H-F-H plate can be obtained.

## 3. Theoretical Solution and Numerical Simulation

### 3.1. Comparison of Theoretical Solution and Numerical Simulation Displacement for H-H-H-H Plate

To obtain the theoretical solution for the H-H-H-H plate, the material parameters of the plate are input into Equation (40). The random generation function for raindrop impacts (Equation (23)) is used to simulate the external force acting on the plate. To solve the nonlinear differential equations governing the system’s vibration and voltage response, a fourth-order Runge–Kutta (RK-4) method was employed. The algorithm was developed and implemented in-house using FORTRAN, rather than relying on commercial software, to allow full control over the simulation parameters and integration process.

**Figure 3:**
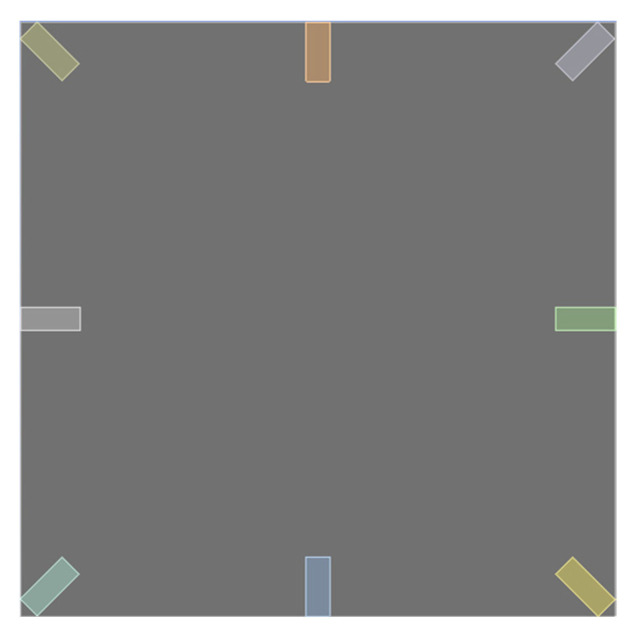
ANSYS simulation of H-H-H-H plate with integrated piezoelectric patches positions.

Finally, the displacement is substituted into the piezoelectric equations (Equations (31)–(38)) to calculate the output voltage of the piezoelectric patches positioned at various locations. Additionally, this study uses the commercial software ANSYS Workbench to verify the analysis. The model consists of piezoelectric patches placed at different positions beneath a square plate made of elastic steel. The positions are divided into the four edges and four corners of the square plate, as shown in Figure 3. Material parameters for elastic steel and the piezoelectric patches are input, and in the DM module, the length and width of the plate are set to 500 mm, with a thickness of 0.15 mm. The “DM model” refers to the DesignModeler module in ANSYS, which is used to create the geometry of the simulation model. In this study, it was used to construct the plate structure with integrated PZT elements, as illustrated in Figure 3. We have clarified this in the revised manuscript. The piezoelectric patch dimensions are set to 50 mm by 20 mm, with a thickness of 0.6 mm.

**Figure 4:**
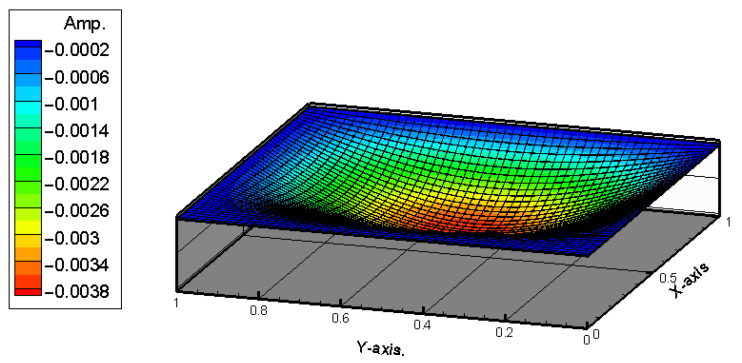
Theoretical prediction of plate displacement due to rain deformation.

**Figure 5:**
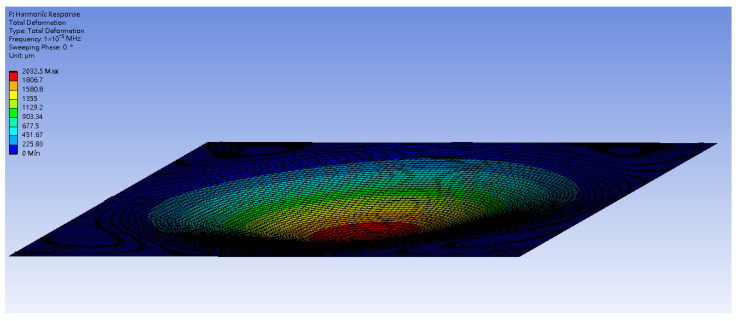
ANSYS Workbench simulation of plate displacement due to rain deformation.

To obtain the theoretical solution, the material parameters of the plate are input into Equation (39). The random generation function for raindrop impacts is used to simulate the external forces acting on the plate. The RK-4 method is then used to solve for the displacement of the plate, incorporating the piezoelectric patches. Ansys and theoretical results provide comparable displacement maps, validating the nonlinear plate model. Figure 4 and Figure 5 show the displacement maps for the H-H-H-H plate. The displacement distributions are similar in both cases, with the maximum displacement occurring at the center of the plate. For a plate with dimensions 1000 mm by 1000 mm, the theoretical prediction for the displacement at the center point is 4.004 mm, while the simulation in ANSYS Workbench for a plate with dimensions 500 mm by 500 mm shows a displacement of 2.0325 mm at the center. After conversion, the error is found to be 1.5%. This confirms that the assumptions and derivations of the nonlinear plate motion equation are accurate. All material properties are based on standard values for structural steel and commercially available PZT-5H piezoelectric patches, as summarized in Table 1.

### 3.2. Comparison of Theoretical Solution and Numerical Simulation Displacement for C-H-F-H Plate

**Figure 6:**
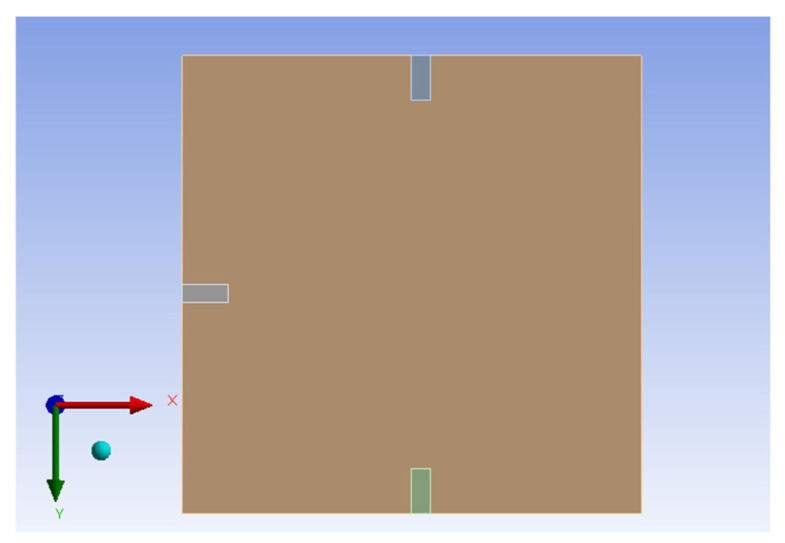
ANSYS simulation of C-H-F-H plate with integrated piezoelectric patches positions for displacement simulation.

**Figure 7:**
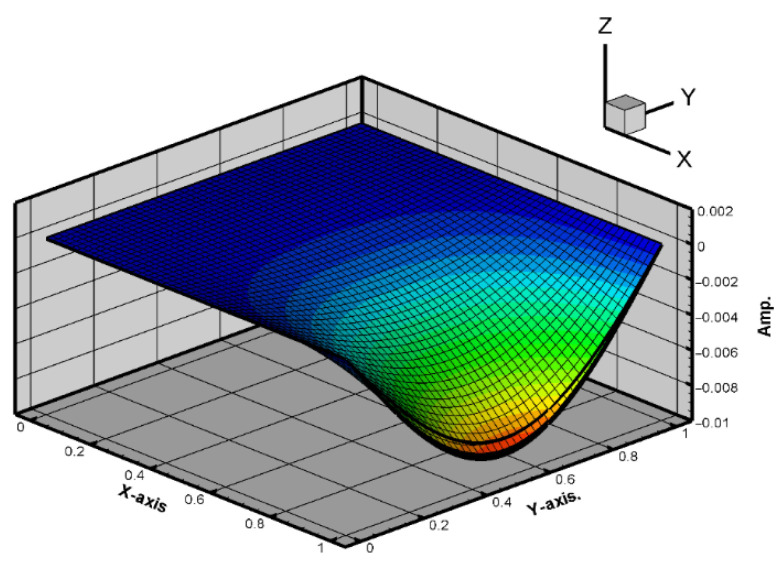
Plate amplitude response under external force disturbance.

**Figure 8:**
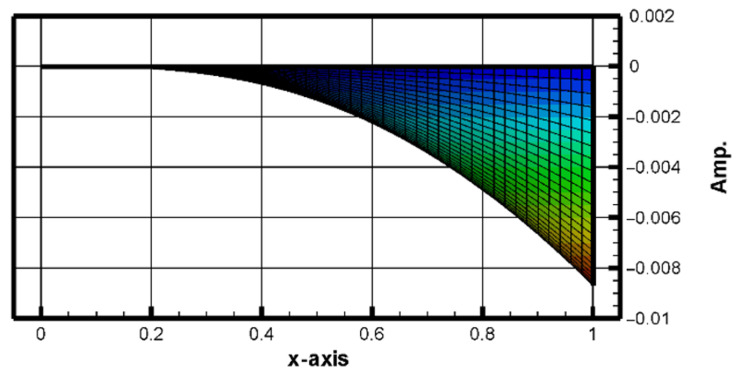
Side view in x-z plane.

Same as H-H-H-H plate, this study also uses the commercial software ANSYS Workbench to verify the C-H-F-H plate displacement under external forces. The model consists of piezoelectric patches placed at different positions beneath a square plate made of elastic steel. These positions are shown in Figure 6. The RK-4 method is used to solve for the displacement of the plate under random external force disturbances. First, Equation (22) is combined with Equation (23), considering only the first mode. The plate size is 1 × 1 (square). The results from the RK-4 numerical method are plotted, as shown in Figure 7 and Figure 8 shows the 2D side view in the *x–z* plane.

This study also uses the ANSYS Workbench to simulate the C-H-F-H plate under random external force disturbances. The boundary conditions are set as follows:

- For the fixed edge of the plate parallel to the *x*-direction, denoted as *x_d_* = 0, *y_d_* = 0, *z_d_* = 0.
- For the hinged edges of the plate parallel to the *y*-direction at *x* = 0 and *x* = 500, the boundary condition is *x_d_* = 0, *y_d_* = 0, *z_d_* = free.

**Figure 9:**
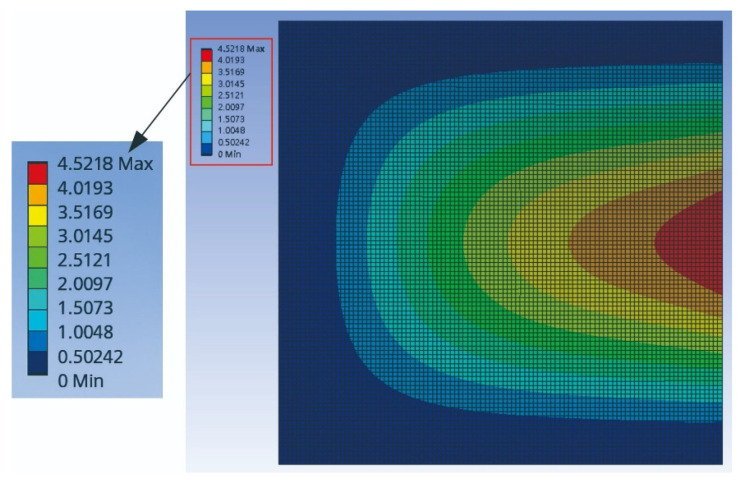
ANSYS simulation of plate amplitude response under external force excitation.

After performing the software simulation, the results are obtained as shown in Figure 9.

The comparison of Figure 7, Figure 8 and Figure 9 shows that the theoretical and numerical simulation amplitudes are very close. For a plate with dimensions 1000 mm by 1000 mm, the theoretical prediction for the maximum displacement is 8.697 mm, while the simulation in ANSYS Workbench for a plate with dimensions 500 mm by 500 mm shows a displacement of 4.5218 mm. After conversion, the error is found to be 2.87%. This confirms that the assumptions and derivations of the nonlinear plate motion equation are accurate.

### 3.3. Comparison of Theoretical and Numerical Simulation Voltage Outputs for H-H-H-H Plate

**Figure 10:**
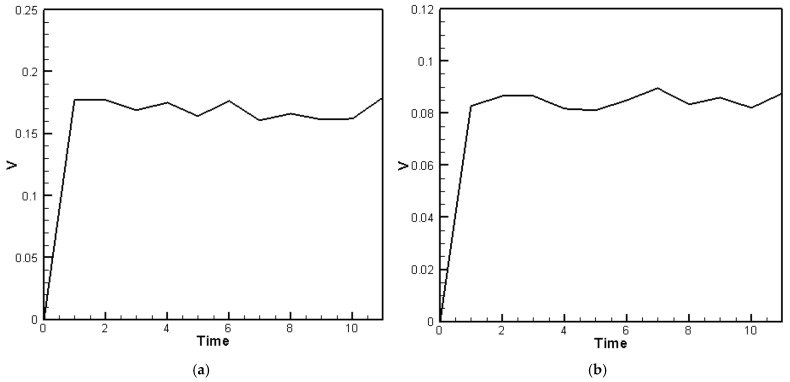
Theoretical voltage response of the H-H-H-H plate, (**a**) piezoelectric patches located at the corners; (**b**) piezoelectric patches located on the edges.

The material parameters of the plate are input into Equation (40), and the random generation function for raindrop impact (Equation (23)) is used to simulate the external force. The displacement of the plate with the piezoelectric patches is then solved using the RK-4 method. The resulting displacement values are substituted into Equations (31)–(38) to compute the voltage output for piezoelectric patches placed at various positions, as shown in Figure 10a,b. Figure 10a presents the theoretical voltage distribution for PZTs located at the corners, while Figure 10b shows the distribution for PZTs located on the edges of the H-H-H-H plate. In the theoretical model, the impact of raindrops is simulated using a set of 10 randomly generated force events over a 10-unit time duration to represent the stochastic nature of rainfall. These events form a representative subset of a continuous random excitation process. The curves in Figure 10a,b represent the averaged results from five independent sets of randomly generated raindrop impact simulations, each consisting of 10 discrete events. This averaging process provides a more statistically representative voltage profile and avoids reliance on any single extreme value.

In the ANSYS simulation, since the structure is not complex, a hexahedral mesh is used for discretization. Hexahedral elements typically produce more accurate results with fewer elements compared to tetrahedral elements. The element size is set to 5 mm, and after meshing, the model of the plate with piezoelectric patches consists of 73,827 nodes and 12,192 elements. The simulation uses APDL to input multiple raindrop impact data, applying force to the plate nodes. The “APDL” stands for Ansys Parametric Design Language, a scripting language used within Ansys Workbench to define custom loading conditions and boundary constraints. In this study, APDL was used to simulate the impact forces generated by raindrops. In simulations with raindrops of various sizes, the voltage output from piezoelectric patches at the corners is higher than that at the sides. The average voltage at the corners is 0.173 V, while at the sides it is 0.138 V. From Figure 10a,b, the theoretical voltage variation obtained using RK-4 does not closely match the results from ANSYS Workbench simulations. The average voltage at the corners, as predicted by RK-4, is 0.17133 V, the ANSYS Workbench simulation gives 0.173 V, resulting in an error of 0.97%. The average voltage at the edges predicted by RK-4 is 0.0851 V, while the ANSYS Workbench simulation gives 0.138 V, with an error of about 38.33%. The average voltage at the corners in the theoretical prediction is approximately 2.0 times higher than that at the edges, while the ANSYS Workbench simulation gives a ratio of 1.25 times. Here is a summary of the results presented in Table 2.

The results show that the power generation voltage at the corners, as predicted by the theoretical model and ANSYS simulation, is higher than that at the hinged edges. However, the error in the voltage at the hinged edges is significantly larger than at the corners. This discrepancy is presumed to arise because the vibration and deformation of the hinged edges are influenced by the adjacent corners, which act as fixed points. Although the theoretical model assumes perfectly hinged boundary conditions, the hinged edges are physically connected to the fixed corners and are therefore partially constrained. ANSYS accounts for this interaction, which results in a greater deviation between the theoretical predictions and the simulation results.

### 3.4. Comparison of Theoretical and Numerical Simulation Voltage Output for C-H-F-H Plate

Based on the theoretical model structure from Equation (22), combined with the external force function (Equation (23)), and the Coulomb forces of the piezoelectric patches at three different positions (Equations (41)–(43)), the following equation can be obtained:

(44) $$\begin{array}{l} \ddot{w}+w_{xxxx}+2w_{xxyy}+w_{yyyy}+G_{1}+G_{2}+G_{3}=-[-12u_{x}w_{xx}-6{w_{x}}^{2}w_{xx}-12\nu (v_{y}w_{xx}\\ +\frac{1}{2}{w_{y}}^{2}w_{xx})-12v_{y}w_{yy}-6{w_{y}}^{2}w_{yy}-12\nu (u_{x}w_{yy}+\frac{1}{2}{w_{x}}^{2}w_{yy})-12(1-\nu )(u_{y}w_{xy}+v_{x}w_{xy}+w_{x}w_{y}w_{xy})]+F(t) \end{array}$$

**Figure 11:**
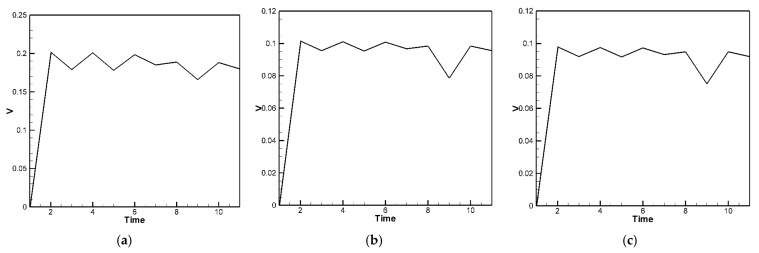
Theoretical voltage response of the C-H-F-H plate over time for three piezoelectric patches, (**a**) at the clamped boundary (*G*_1_); (**b**) at the hinged boundary *y* = 0, (*G*_2_); (**c**) at the hinged boundary *y* = 1, (*G*_3_).

For the C-H-F-H plate, the RK-4 method is used to compute the theoretical voltage output under similar random excitations. As with the H-H-H-H case, the voltage responses for the three piezoelectric patches are averaged over five random datasets and plotted in Figure 11a–c. The results demonstrate effective energy harvesting performance, particularly along the clamped boundary, where the energy conversion is more efficient than on the hinged edges.

**Figure 12:**
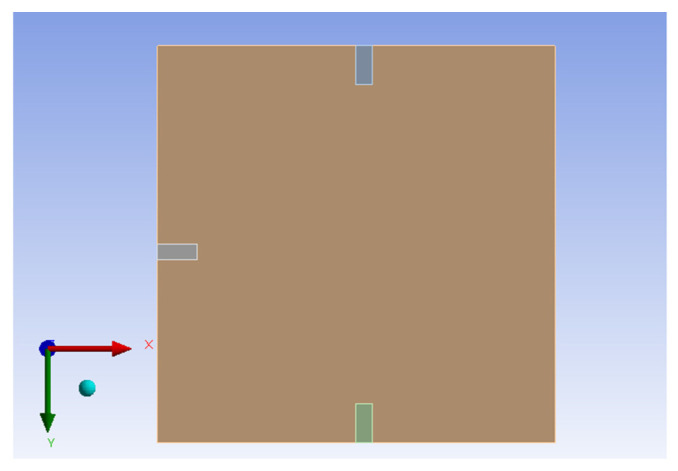
ANSYS simulation of C-H-F-H plate with integrated piezoelectric patches positions for voltage simulation.

Additionally, this study also uses the ANSYS Workbench combined with the built-in PZT module, to calculate the simulated voltage output from the three piezoelectric patches. The three piezoelectric patches are integrated on the *x*-*y* plane of the plate. The 2D diagram is shown in Figure 12.

Hexahedral meshing is used for grid division. Since the piezoelectric patches are much smaller than the plate, hexagonal cubes (5 × 5 × 1 (mm)) are selected for higher simulation accuracy. The boundary conditions of the plate are set based on the real model framework. Specifically, the plate is fixed along the *x*-direction (*y* = 0), and the boundary conditions are set accordingly; the two hinged edges of the plate are along the *y*-direction (*x* = 0 and *x* = 500), with the boundary conditions set for those as well. First, modal analysis is performed on the plate with piezoelectric patches using ANSYS Workbench to determine its first mode natural frequency. Then, random external forces are applied, where the random force matrices are generated in the same manner as in the previous sections (using ten different random number matrices, 100 × 100 matrix *R*(*i*, *j*)), and applied to the *x–y* plane nodes of the plate. After simulation in ANSYS, the output voltage for the three piezoelectric patches is calculated. Table 3 summarizes the theoretical and simulated voltage values for the three piezoelectric patches.

The results show that the power generation voltage at the clamped edge, as predicted by the theoretical model and ANSYS simulation, is higher than that at the two hinged edges. However, the error in the power generation voltage at the hinged edges is much larger than that at the clamped edge. This is presumed to be due to the fact that the vibration and deformation of the hinged edges are influenced by the clamped edge. While the theoretical model assumes perfectly hinged condition, the ends of the hinged edges are actually connected to the clamped edge, and thus are affected to some extent by the clamping. ANSYS, however, is able to account for this effect, leading to a larger discrepancy between the theoretical prediction and the simulation.

## 4. Experimental Setup and Discussion

**Figure A1:**
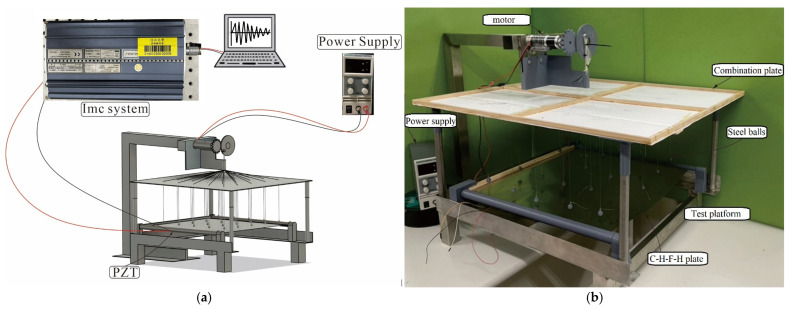
Experimental setup (C-H-F-H configuration) (**a**) system schematic; (**b**) photograph.

**Figure A2:**
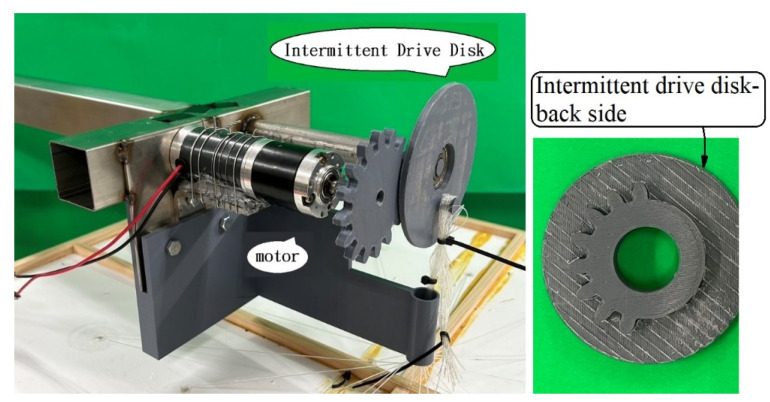
Motor and ‘intermittent drive disk’.

**Figure A3:**
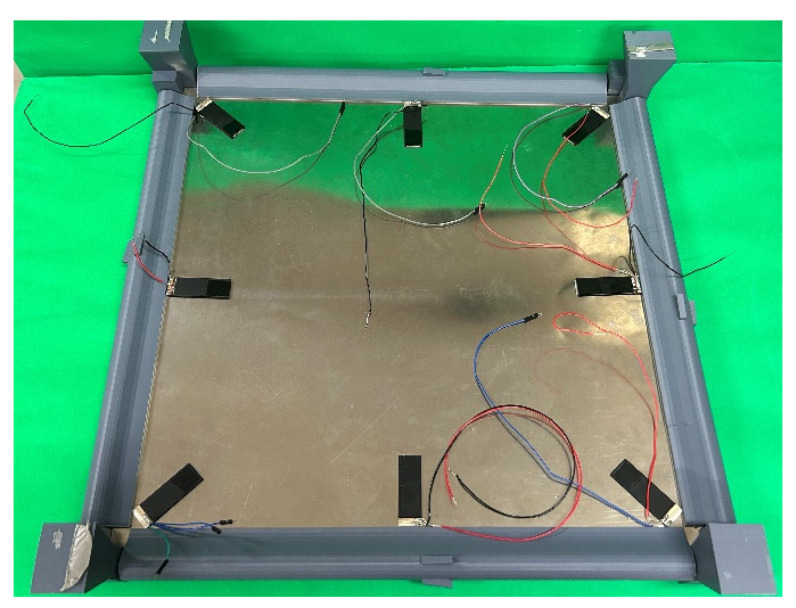
Rear view of H-H-H-H elastic steel plate.

**Figure A4:**
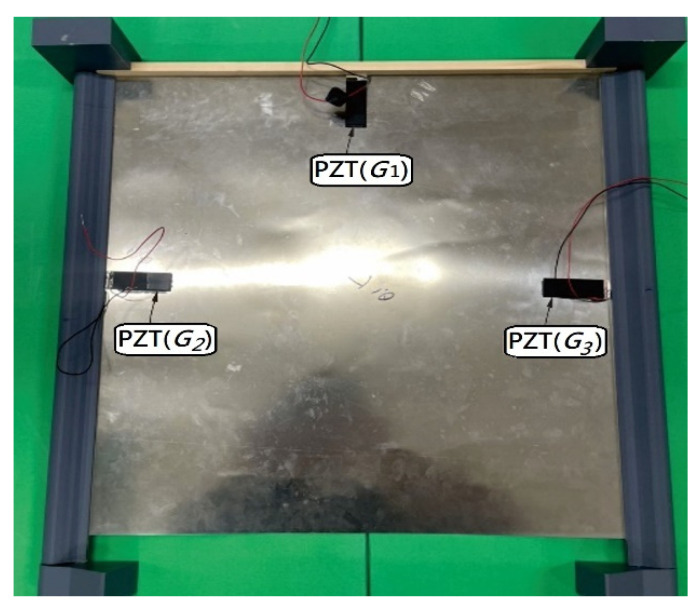
Rear view of C-H-F-H elastic steel plate.

To assess the feasibility of the proposed Rain Energy Harvester (REH) system and evaluate the accuracy of the theoretical and simulation models, a simple experimental setup was developed. A steel plate was used to simulate the vibrating plate in both C-H-F-H and H-H-H-H boundary configurations. For the C-H-F-H configuration, the plate was supported by a frame structure. The clamped edge was fixed using two wooden blocks and C-clamps. Hinged boundaries were simulated by placing a circular pipe beneath the steel plate and inserting it onto two rods fixed to the wooden blocks (see Appendix D, Figure A1). For the H-H-H-H case, four rods were positioned at the corners of the frame, and a circular pipe beneath the plate was inserted onto these rods to simulate fully hinged edges. To replicate the random impacts of falling raindrops, steel balls were used as the excitation source. A motor-driven intermittent drive mechanism (Appendix D, Figure A2) was employed to lift and release the steel balls in a controlled and repeatable manner. Dozens of steel balls were tied with fine strings and arranged via a combination plate above the vibrating surface (Appendix D, Figure A3 and Figure A4). Piezoelectric patches were placed at predetermined locations beneath the plate. A laser displacement sensor measured plate deflection, and voltage output was recorded using an imc data acquisition system (imc© system, CS-5008-1, TÜV Rheinland, Cologne, Germany). This experimental procedure not only validates the theoretical predictions but also evaluates the system’s performance under real operating conditions.

### 4.1. Experimental Steps

1. The elastic steel plate is fixed, and piezoelectric patches are installed on its underside.
2. The excitation frequency is controlled via a motor connected to an intermittent drive disk.
3. The motor, mounted on a C-shaped bracket with an attached gear, drives the semi-toothed intermittent drive disk (Appendix D, Figure A2). The gear lifts the steel balls to the top of the disk. When reaching the gearless portion, the gear disengages, allowing the balls to fall freely onto the plate—simulating raindrop impacts. The cycle repeats automatically.
4. The imc^©^ system records displacement and voltage signals, and Fourier analysis is used to evaluate frequency response and energy output.
5. PZT patches are placed on the reverse sides of both H-H-H-H and C-H-F-H plates at the designated test positions.
6. Experimental data are compared with simulation results to validate the accuracy of the proposed nonlinear model.

### 4.2. Results and Discussion

**Figure 13:**
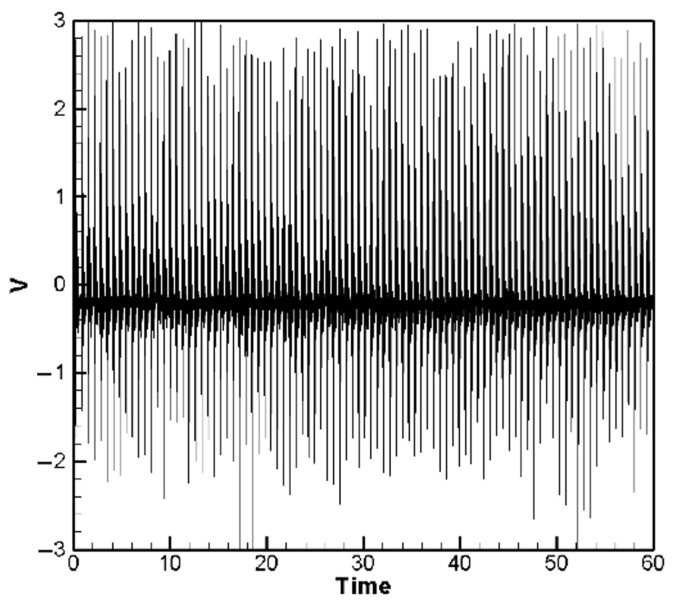
Experimental voltage output of H-H-H-H Plate (corner PZTs).

**Figure 14:**
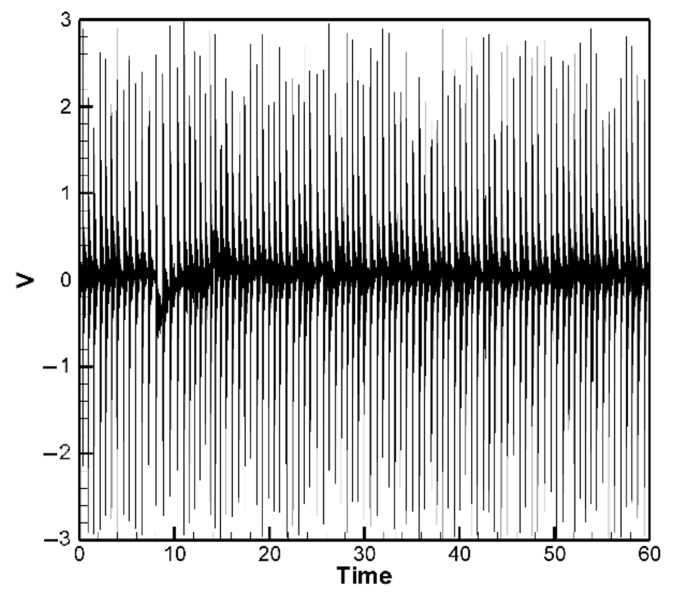
Experimental voltage output of C-H-F-H plate (clamped edge PZT).

The experimental results for the H-H-H-H and C-H-F-H cases are shown in Figure 13 and Figure 14, respectively. Figure 13 presents the experimental voltage output of the H-H-H-H plate for PZTs located at the corners, with the root mean square (RMS) voltage yielding an average of 0.53 V as shown in Table 4. Figure 14 shows the experimental voltage output of the C-H-F-H plate for clamped–mounted PZTs (*G*_1_ position), with the RMS voltage averaging 0.50 V as shown in Table 5. A comparison of these experimental results with theoretical predictions and ANSYS simulations is summarized in Table 4 and Table 5. By comparing the experimental results with the theoretical and numerical simulation results of the H-H-H-H and C-H-F-H plates, it was found that the experimental values were generally higher than the theoretical and numerical simulation values (Table 4 and Table 5). The noticeable discrepancies in Table 4 and Table 5, particularly for the edge-mounted piezoelectric patches, are primarily attributed to the complex boundary interactions—especially in the both H-H-H-H and C-H-F-H configurations, where the combination of clamped and hinged edges introduces nonlinear constraints that are challenging to model precisely in the theoretical formulation. Additionally, other physical factors—such as the slapping force exerted by raindrop impacts on the piezoelectric elements—contribute to deviations between theoretical predictions and experimental results. These effects are difficult to capture fully without refined modeling. We discuss this slapping force correction and its role in reducing error in a later section of the manuscript.

It is noted that in Figure 10 and Figure 11, the theoretical model simulates the impact of raindrops using a set of 10 randomly generated force events to represent the stochastic nature of rainfall. These events serve as a representative subset of a continuous random excitation process. The corresponding voltage responses are calculated from the resulting plate displacements using the piezoelectric coupling equations. This process is repeated for five different sets of random force inputs, and the resulting voltage outputs are averaged to obtain a representative response. In contrast, the experimental setup uses a continuous stream of steel balls to simulate rainfall, resulting in a denser voltage output over time (Figure 13 and Figure 14). For extended durations (e.g., over 60 s), repeating the random excitation process in simulation with more samples would yield a voltage profile statistically similar to the experimental result. Therefore, the theoretical results are considered sufficient to demonstrate the system’s performance trends and energy harvesting capability. Also notably, the experimental output voltage of the PZT elements installed at the hinged edges were significantly higher than the theoretical values. Upon closely observing the experimental conditions, it was discovered that when the simulated raindrop steel balls fell, they not only caused the elastic plate to deform—leading to deformation of the PZT elements beneath and subsequent power generation—but also exerted an impact force directly on the PZT elements. This additional factor allowed the PZT to convert more electrical energy. Furthermore, although the hinged edges of the plate were simulated using a rotational axis during the experiment, the elastic steel sheet remained a rigid material. Whether the adjacent edges of the hinged boundary were clamped or also hinged, the hinged structure, while allowing the plate or PZT to rotate freely, was still constrained by the adjacent edges. This limitation was particularly evident in both the cases of the H-H-H-H and C-H-F-H plates, where a large portion of the structure was influenced by the clamped or cornered boundary. Consequently, it was not feasible to set the boundary as completely “hinged” in the simulation. The primary goal of this study is to verify the feasibility of the proposed concept; therefore, a detailed simulation of boundary conditions is beyond the scope of this paper. However, in terms of the impact of simulated raindrops impacting the PZT and applying additional forces, this study refers to the work of Wang et al. [35,36,37], who have investigated the effects of impulsive forces on piezoelectric elements in a series of studies. Based on Wang et al. [35,36,37], we can assume that the additional force exerted on the PZT by raindrop impacts can be expressed as follows:

(45) $$\hat{F}=\rho A\ddot{\bar{w}}({\bar{x}}_{P},\bar{t})\delta (\bar{t}-\bar{T}),\;\bar{t}>0$$

where *x_P_* is the free-end position of the PZT, ρ is the density of the plate, *A* represents the area of the plate, *T* is the slapping period and $\delta \left(\bar{t}\right)$ is the Dirac delta function, dividing the above equation by $\frac{EI}{\rho Al^{3}}$ yields the dimensionless slapping force:

(46) $$\bar{F}=\ddot{w}(x_{P},t)\delta (t-T)$$

Equation (46) can be considered as an external force correction term for the PZT.

In the case of the H-H-H-H plate, adding Equation (46) to Equation (40) yields the following equation:

(47) $$\begin{array}{l} w_{\alpha \alpha \alpha \alpha }+2w_{\alpha \alpha \beta \beta }+w_{\beta \beta \beta \beta }=\tilde{F}-\gamma \ddot{w}-\zeta \dot{w}+\varepsilon \left\{12u_{\alpha }w_{\alpha \alpha }+6w_{\alpha }^{2}w_{\alpha \alpha }+12\nu \left(v_{\beta }w_{\alpha \alpha }+\frac{1}{2}w_{\beta }^{2}w_{\alpha \alpha }\right)\right.\\ +12v_{\beta }w_{\beta \beta }+6w_{\beta }^{2}w_{\beta \beta }+12\nu \left(u_{\alpha }w_{\beta \beta }+\frac{1}{2}w_{\alpha }^{2}w_{\beta \beta }\right)\left.+12(1-\nu )\left(u_{\beta }w_{\alpha \beta }+v_{\alpha }w_{\alpha \beta }+w_{\alpha }w_{\beta }w_{\alpha \beta }\right)\right\}+P+\bar{F} \end{array}$$

**Figure 15:**
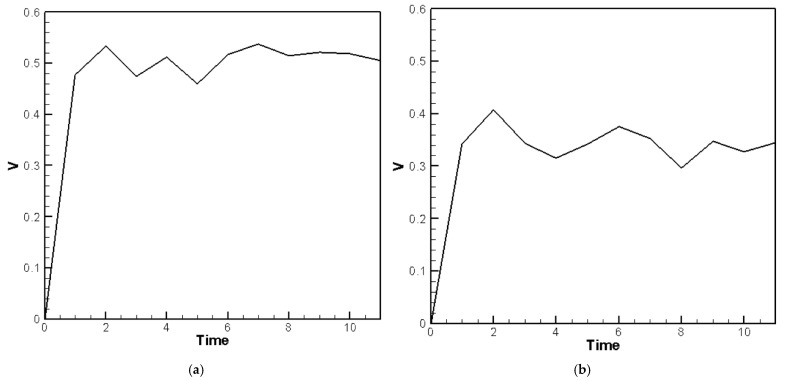
The H-H-H-H plate theoretical average voltage-time response with slapping forces (**a**) at the four corners; (**b**) at the four edges.

The fourth-order Runge–Kutta numerical method is used to calculate the theoretically generated voltage of the plate under random external excitation and slapping forces. The voltage-time response graphs for the piezoelectric patches at the four corners and four edges are plotted, as shown in Figure 15a,b. These graphs represent the output voltage resulting from five sets of randomly generated raindrop impact forces, with each set processed by calculating the average results to obtain a representative voltage response. Furthermore, we observed that during the raindrop impact process, the force exerted on the plate is quite significant. This can be corrected using our theoretical model. However, in the ANSYS simulation, the raindrop is directly modeled to strike the plate, causing deformation, and the built-in piezoelectric conversion function is then used to simulate the output. This approach does not accurately account for the additional impact force of the raindrop. As a result, there is a large discrepancy between the ANSYS simulation and experimental results. Therefore, the ANSYS simulation results are excluded from further discussion. Although ANSYS is a highly capable simulation platform that supports complex modeling of heterogeneous piezoelectric materials and structures, including porosity and multiphase composites, its standard piezoelectric modules do not natively account for stochastic or impact-type excitations such as transient slapping forces caused by raindrop impacts. In this study, the slapping force is treated as a localized, time-dependent impact, which is more conveniently handled through a customized theoretical model rather than within the default ANSYS environment. Therefore, the purpose of using ANSYS in this work is primarily to validate the deformation trends and voltage response under deterministic loading conditions. The slapping-force correction is implemented in the theoretical model to better capture the dynamic nature of rain-induced impacts, and the simulation results are interpreted accordingly.

The comparison and error between theory and experiment are shown in Table 6. As shown in Table 6, for the piezoelectric patches located at the four corners, the results indicate that the theoretical and experimental energy conversion values are close, with the error reduced from 67.7% to 3.88%. This confirms that after including the slapping force, the discrepancy between theoretical and experimental energy conversion has been effectively minimized. However, the piezoelectric patches located along the four edges are still affected by the combined fixed-hinged boundary conditions, resulting in a remaining 32% error between theory and experiment. This issue stems from the basic assumptions and would require correction of the theoretical boundary conditions, which is beyond the scope of this study. Nevertheless, the error has been significantly reduced, demonstrating that the proposed correction model is feasible.

For the C-H-F-H case, by incorporating Equation (46) into Equation (44), the following equation is obtained:

(48) $$\ddot{w}+w_{xxxx}+2w_{xxyy}+w_{yyyy}+G_{1}+G_{2}+G_{3}=-[-12u_{x}w_{xx}-6{w_{x}}^{2}w_{xx}-12\nu (v_{y}w_{xx}+\frac{1}{2}{w_{y}}^{2}w_{xx})-12v_{y}w_{yy}-6{w_{y}}^{2}w_{yy}\;-12\nu (u_{x}w_{yy}+\frac{1}{2}{w_{x}}^{2}w_{yy})-12(1-\nu )(u_{y}w_{xy}+v_{x}w_{xy}+w_{x}w_{y}w_{xy})]+F(t)+\bar{F}(t)$$

**Figure 16:**
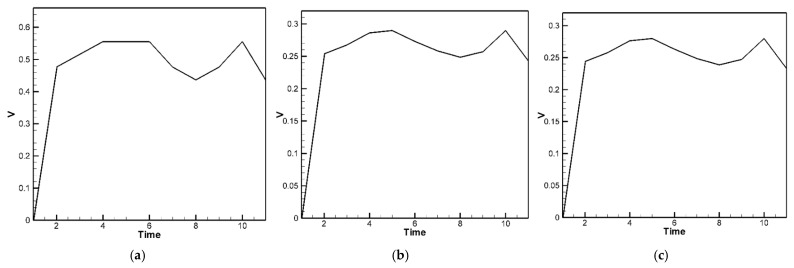
The C-H-F-H plate theoretical voltage-time response plots with slapping forces (**a**) at *G*_1_; (**b**) at *G*_2_; (**c**) at *G*_3_.

Next, the fourth-order Runge–Kutta numerical method is used to calculate the theoretical generated voltage of the plate under random external excitation and slapping forces. Figure 16a–c present the voltage-time response curves for the three piezoelectric patches in the C-H-F-H configuration. These results are based on five independently simulated sets of random raindrop impacts, where each set’s voltage output is averaged to produce a representative and smoothed voltage profile. Again, the Ansys simulation results are excluded from further discussion. The comparison and error between theory and experiment are shown in Table 7. As shown in Table 7, for the piezoelectric patches located at the clamped edge (*G*_1_), the results indicate that the theoretical and experimental energy conversion values are close, with the error reduced from 66.51% to 1.84%. This confirms that after including the slapping force, the discrepancy between theoretical and experimental energy conversion has been effectively minimized. However, the piezoelectric patches located along the hinged edges (*G*_2_ and *G*_3_) are still affected by the combined clamped–hinged boundary conditions, resulting in a remaining 29~34% error between theory and experiment. Again, this issue arises from fundamental assumptions and would require modifications to the theoretical boundary conditions, which are beyond the scope of this study. Nevertheless, the error has been significantly reduced, demonstrating the feasibility of the proposed correction model in both the H-H-H-H and C-H-F-H cases.

## 5. Power Management System Design

Having validated the proposed rain energy harvesting system through theoretical modeling, numerical simulation, and experimental verification, it is essential to explore its potential deployment in real-world environments. The following section outlines practical applications, scalability considerations, and integration strategies, particularly in rain-prone and energy-deficient regions. These insights directly reflect on the system’s practical viability and long-term relevance.

**Figure 17:**
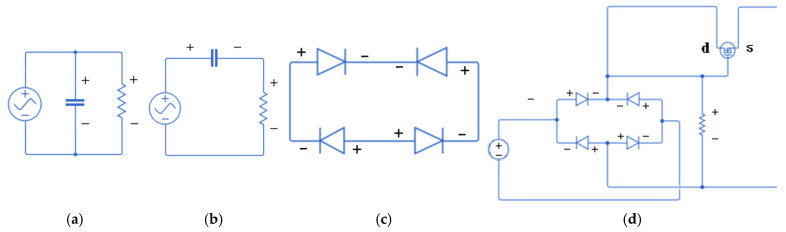
(**a**) Equivalent circuit for piezoelectric current; (**b**) Equivalent circuit for piezoelectric voltage; (**c**) Full bridge rectifier composed of diodes; (**d**) Full bridge rectifier connected with N-channel MOSFET.

According to the general principle of battery charging, the charging voltage must exceed a certain threshold to allow continuous energy flow into the battery. If the charging voltage is too low, it may instead cause the battery to discharge. To achieve effective energy storage, this study designs a power management system with the goal of enabling the proposed design to function effectively and fulfill the purpose of energy storage. In the power management system, the circuit simulation is carried out using Simulink (Figure 17a–d). The equivalent circuits for current and voltage behavior of the piezoelectric patches are shown in Figure 17a,b. Since the output of piezoelectric patches is alternating current (AC), it must be rectified into direct current (DC) for practical use. This is achieved using a full-bridge rectifier composed of diodes (Figure 17c). An N-channel MOSFET (NMOS) is then integrated to conduct the current more effectively, especially under higher output voltages, forming the basic unit of the circuit (Figure 17d).

For the H-H-H-H plate, four such units (each consisting of a piezoelectric patch, rectifier, and NMOS circuit) are connected in parallel. For the C-H-F-H plate, three units are connected in parallel. Parallel connection is chosen over series due to its higher energy harvesting efficiency and its ability to mitigate mismatched voltages across different patches. To ensure the MOSFET operates correctly, a resistor is included to maintain the gate-source voltage above the threshold level. A boost (step-up) converter is added after the parallel circuits to stabilize the output voltage for powering external loads. This configuration ensures consistent voltage during vibrations and prevents energy loss when connecting piezoelectric patches of varying output voltages.

**Figure 18:**
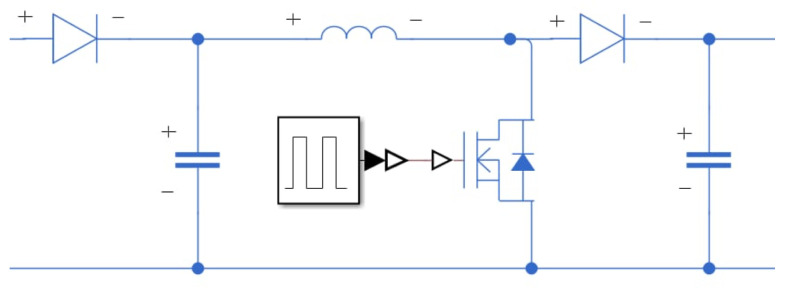
Boost converter circuit diagram.

**Figure 19:**
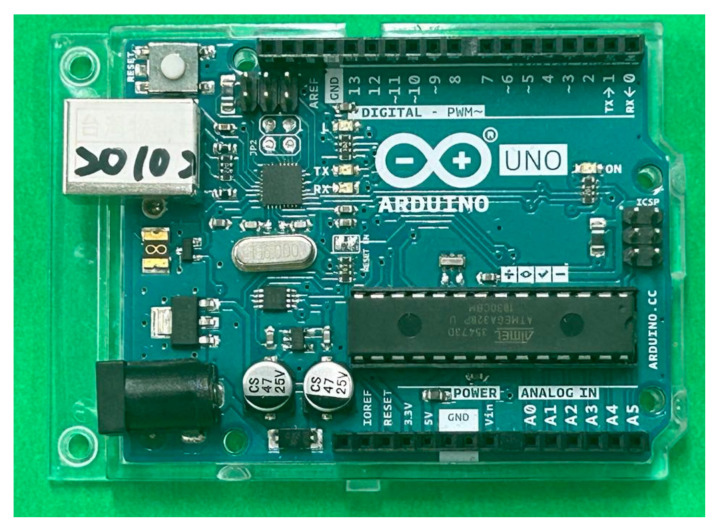
A photograph of Arduino hardware setup.

The complete circuit—consisting of four parallel-connected rectifier-NMOS units followed by a boost converter—is illustrated in Figure 18. The switching element in the boost converter is controlled using an Arduino UNO (Figure 19). In this study, the vibration frequency (representing rainfall) is set at approximately 2 Hz, with a moderate rain intensity. The relation between input voltage (U_in_), output voltage (U_out_), and duty cycle (*q*) is expressed as U_out_(1 − *q*) = U_in_, where *q* represents the duty cycle. This formula is used to calculate the duty cycle and set the switching timing. The Arduino’s pin 7 controls the NMOS switch, while pin A0 monitors the output voltage of the piezoelectric array. Based on the voltage readings, the Arduino adjusts the switching frequency to optimize converter performance. The Arduino UNO sends a signal to the NMOS to control the switch. When the NMOS is turned on, the circuit becomes conductive, and the NMOS acts like a ground wire. The circuit then forms a loop, with current flowing clockwise through the inductor. The inductor converts electrical energy into a magnetic field to store energy. The left side of the inductor is positive. When the NMOS is turned off, the inductor current cannot change abruptly, so the current continues to flow to the right. This allows the voltage from the power source, which is the piezoelectric patch, to be in series with the inductor voltage.

**Figure 20:**
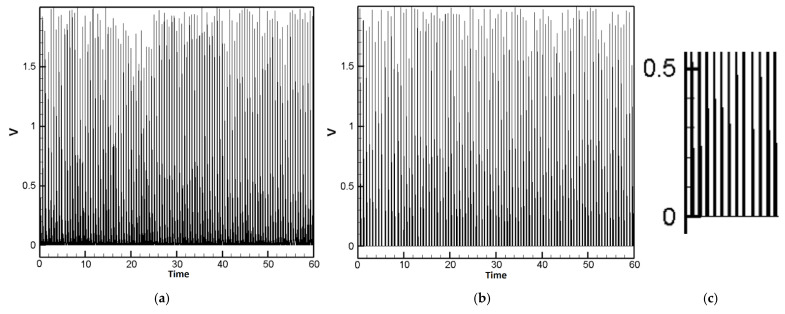
H-H-H-H case rectified voltage waveform, (**a**) before NMOS (PM) switching; (**b**) after NMOS switching (PM); (**c**) zoomed-in view of a segment from Figure 20b.

For the case of H-H-H-H plate, the piezoelectric patch used in the experiment outputs an AC signal with an average voltage of approximately 0.53 V and an average current of 0.01 mA. After passing through a full-wave bridge rectifier composed of Schottky diodes, due to the typical voltage drop of about 0.12 V across the Schottky full-wave bridge, the resulting DC output has an average voltage of approximately 0.41 V (Figure 20a). Two piezoelectric patches (corner and edge) are connected in parallel using an NMOS. After rectification, the positive terminal is connected to both the “Gate” and “Drain” of the NMOS, while the negative terminal is grounded. When the gate voltage exceeds the threshold voltage of 0.2 V, the NMOS turns on, and the output voltage equals the input voltage. When the gate voltage is below the threshold, the NMOS turns off and the output voltage drops to zero. As shown in Figure 20b, the vertical axis represents voltage and the horizontal axis represents time. The average output voltage after NMOS switching is approximately 0.38 V. Figure 20c is a zoomed-in view of a portion of Figure 20b, showing that voltages below 0.2 V are blocked. Since this design blocks voltage flow below 0.2 V, it may reduce the average voltage, but it helps prevent excessive voltage differences between parallel-connected piezoelectric patches, thereby reducing current loss, improving efficiency, and providing a more stable output voltage.

**Figure 21:**
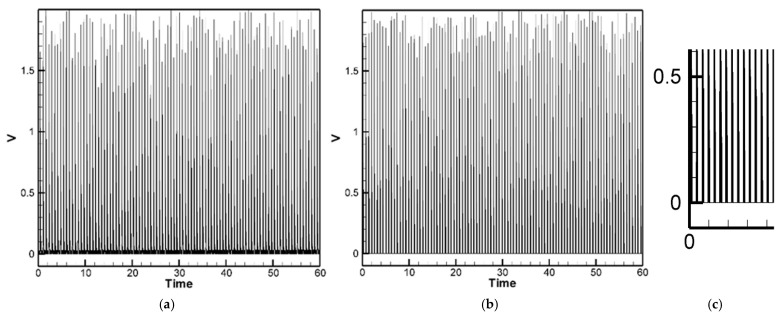
C-H-F-H case rectified voltage waveform, (**a**) before NMOS (PM) switching; (**b**) after NMOS switching (PM); (**c**) zoomed-in view of a segment from Figure 21b.

For the case of C-H-F-H plate, the same as H-H-H-H plate, piezoelectric patch used in the experiment outputs an AC signal with an average voltage of approximately 0.50 V and an average current of 0.01 mA. After passing through a full-wave bridge rectifier composed of Schottky diodes, due to the typical voltage drop, the resulting DC output has an average voltage of approximately 0.38 V (Figure 21a). Three piezoelectric patches (*G*_1_, *G*_2_ and *G*_3_) are connected in parallel via an NMOS transistor. After rectification, when the gate voltage exceeds the threshold of 0.2 V, the NMOS turns on, allowing the output voltage to follow the input voltage. As shown in Figure 21b, the average output voltage after NMOS activation is approximately 0.34 V. Figure 21c presents a zoomed-in view of a selected portion of Figure 21b, clearly illustrating that input voltages below 0.2 V are effectively blocked.

This power management system actively monitors the energy output from each piezoelectric unit and dynamically adjusts to select the most efficient energy source. It enables effective vibration energy harvesting, stable DC conversion, and reliable power delivery for downstream loads. Although the measured average current output from the proposed system is relatively low (approximately 0.01 mA), the ability to achieve a stable voltage output through the implemented power management circuit represents a significant milestone in validating the theoretical framework. This outcome confirms that the rain-induced impact model, including the slapping force correction, is both feasible and functionally effective. The system’s capability to stabilize voltage under randomly distributed raindrop excitations serves as a critical proof-of-concept for real-world energy harvesting scenarios. While the harvested current may not yet be sufficient for direct battery charging, it is already suitable for powering ultra-low-power devices, such as environmental sensors or wireless communication modules operating on intermittent duty cycles. Future enhancements—such as increasing the number of piezoelectric elements, optimizing plate geometry and material, or incorporating energy storage components like supercapacitors—can further improve the energy output and broaden the application scope. Therefore, the success of voltage regulation under stochastic excitation provides a solid foundation for the continued advancement of rain-based vibration energy harvesting systems.

## 6. Conclusions

This study presents a rain-induced vibration energy harvesting system (REH) using nonlinear thin plates with integrated piezoelectric elements. Two structural configurations were investigated: a fully hinged plate (H-H-H-H) and a clamped–hinged–free–hinged plate (C-H-F-H). The nonlinear plate behavior was modeled using Hamilton’s principle combined with classical Kirchhoff plate theory and von Kármán-type geometric nonlinearity. The governing equations were solved using the fourth-order Runge–Kutta method under random impact forces simulating raindrop excitation.

A key contribution of this study is the inclusion of slapping forces, representing localized raindrop impacts. Theoretical results that incorporate slapping forces show significantly improved agreement with experimental measurements, reducing the voltage prediction error from 67.7% to 3.88% for corner-mounted piezoelectric patches. In contrast, edge-mounted patches remain affected by dual boundary interactions, resulting in a residual error of approximately 32%, which is acknowledged as a modeling limitation beyond the current study’s scope.

Comparative analysis between theoretical simulations and ANSYS results further validates the nonlinear plate model under deterministic loading. While ANSYS effectively simulates structural deformation, its standard framework does not account for transient, stochastic slapping forces, highlighting the necessity of a customized theoretical approach.

Additionally, a power management circuit was integrated to achieve stable voltage output from the harvested vibration energy. Although the current output remains relatively low (~0.01 mA), the successful rectification and voltage stabilization confirm the system’s functional feasibility and lay the groundwork for future energy storage enhancements.

Overall, the proposed REH system demonstrates promising potential for deployment in rain-prone environments, with applications in self-powered sensors, canopies, tents, and hybrid solar–rain energy systems. Future work will focus on extending excitation duration, increasing piezoelectric density, and optimizing structural design to improve energy output and broaden practical applications.

Future research will aim to improve the realism and effectiveness of the proposed energy harvesting system. First, the excitation model will be extended to cover longer durations and more frequent raindrop events to better simulate continuous rainfall conditions. Second, the refinement of the theoretical model—particularly in capturing the complex interactions at clamped–hinged boundaries—will be undertaken to improve accuracy. Lastly, integration with energy storage components, such as supercapacitors or low-power storage modules, will be explored to develop a fully functional and autonomous rain energy harvesting system for practical applications.

## Figures and Tables

**Table 1:** Material and geometric parameters used in the numerical model.

Component	Parameter	Value	Unit
Structural Steel Plate	Young’s modulus	2.0 × 10^11^	Pa
	Poisson’s ratio	0.3	
	Length	500	mm
	Width	500	mm
	Thickness	0.15	mm
PZT Actuator	Part number	401,010	
	Dimensions (L × W × T)	50.0 × 20.0 × 0.60	mm
	Capacitance	170,000	pF
	Resonant frequency	65	Hz
ANSYS mesh size	5 mm		
Element type	Structural	SOLID186	
Boundary Conditions	Plate edges	H-H-H-H/C-H-F-H	
External Force	Type	Random impact at (*i*, *j*)	

**Table 2:** Comparison of theoretical and ANSYS simulation errors for PZTs installed at the corners and sides.

	Theory (RK-4)	ANSYS Simulation	Error (%)
Average corners voltage (V)	0.17133	0.173	0.97%
Average edges voltage (V)	0.0851	0.138	38.33%

**Table 3:** Theoretical vs. simulated maximum voltage values for piezoelectric patches.

	Theory (RK-4)	ANSYS Simulation	Error (%)
PZT ($G_{1}$, V)	0.168182	0.164958	2.34%
PZT ($G_{2}$, V)	0.0846	0.116571	27.44%
PZT ($G_{3}$, V)	0.0816	0.118725	31.29%

**Table 4:** Comparison and error between theory, simulation, and experiment (H-H-H-H).

Average Voltage (V)	Theory (RK-4)	Ansys Simulation	Expt.	Theory Experiment Error (%)	Simulation/Experiment Error (%)
Corner	0.171328	0.173	0.53	67.7%	67.35%
Edge	0.0851	0.138	0.51	83.3%	72.94%

**Table 5:** Comparison and error between theory, simulation, and experiment (C-H-F-H).

Average Voltage (V)	Theory (RK-4)	Ansys Simulation	Expt.	Theory Experiment Error (%)	Simulation/Experiment Error (%)
PZT ($G_{1}$)	0.168182	0.164958	0.502245	66.51%	67.17%
PZT ($G_{2}$)	0.0846	0.116571	0.358073	76.37%	67.45%
PZT($G_{3}$)	0.0816	0.118725	0.374363	78.20%	68.27%

**Table 6:** Comparison and error between theory and experiment (H-H-H-H).

Average Voltage (V)	Theory with Slap	Expt.	Error (%)
Corner	0.5094	0.53	3.88%
Edge	0.344912	0.51	32%

**Table 7:** Comparison and error between theory and experiment (C-H-F-H).

Average Voltage (V)	Theory with Slap	Expt.	Error (%)
Clamped ($G_{1}$)	0.493	0.502245	1.84%
Hinged ($G_{2}$)	0.25194	0.358073	29.65%
Hinged ($G_{3}$)	0.24339	0.374363	34.98%

## Data Availability

No new data was created.

## Appendix A

A. Nonlinear equation for H-H-H-H plate

Considering a square simply supported plate (H-H-H-H plate) with side lengths *a* and *b* where *a* = *b*, the boundary conditions are as follows:

(A1) $$w\left(x,0,t\right)=w\left(x,b,t\right)$$

(A2) $$w\left(0,y,t\right)=w\left(a,y,t\right)$$

(A3) $$\frac{\partial^{2}w}{\partial x^{2}}(0,y,t)=\nu \frac{\partial^{2}w}{\partial y^{2}}(a,y,t)$$

(A4) $$\frac{\partial^{2}w}{\partial x^{2}}(x,0,t)=\nu \frac{\partial^{2}w}{\partial y^{2}}(x,b,t)$$

Using the boundary conditions from Equations (A1)–(A4), to ensure that Equation (13) only contains a representation of *w*, it is necessary to first derive the equations of motion for *u* and *v*. This involves assuming the vibration mode of the plate as follows:

(A5) w=η(τ)sinmπαsinnπβ+ξ(τ)sinnπαsinmπβ

In which, η(τ) and ξ(τ) are the time-dependent modal functions, and α, β are the dimensionless spatial coordinates. Equation (A5) represents the assumed mode shape for the H-H-H-H plate. By substituting Equation (A5) into the nondimensional forms of Equations (7) and (8), the deformation equations in the *u*- and *v*-directions can be obtained as follows.

(A6) $$\begin{array}{l} u_{\alpha \alpha }+\frac{1}{2}\left(1+\nu \right)v_{\alpha \beta }+\frac{1}{2}\left(1-\nu \right)u_{\beta \beta }=\frac{1}{4}\pi^{3}\eta^{2}m\left(m^{2}-\nu n^{2}\right)\sin2m\pi \alpha +\frac{1}{4}\pi^{3}\xi^{2}n\left(n^{2}-\nu m^{2}\right)\sin2n\pi \alpha \\ -\frac{1}{4}\pi^{3}\eta^{2}m\left(m^{2}+n^{2}\right)\sin2m\pi \alpha \cos2n\pi \beta -\frac{1}{4}\pi^{3}\xi^{2}n\left(m^{2}+n^{2}\right)\sin2n\pi \alpha \cos2m\pi \beta \\ +\pi^{3}\eta \xi n\left[m^{2}+\frac{1}{2}\left(1-\nu \right)n^{2}\right]\sin m\pi \alpha \cos n\pi \alpha \sin m\pi \beta \sin n\pi \beta \\ +\pi^{3}\eta \xi m\left[n^{2}+\frac{1}{2}\left(1-\nu \right)m^{2}\right]\cos m\pi \alpha \sin n\pi \alpha \sin m\pi \beta \sin n\pi \beta \\ -\frac{1}{2}\left(1+\nu \right)mn^{2}\pi^{3}\eta \xi \sin m\pi \alpha \cos n\pi \alpha \cos m\pi \beta \cos n\pi \beta -\frac{1}{2}\left(1+\nu \right)m^{2}n\pi^{3}\eta \xi \cos m\pi \alpha \sin n\pi \alpha \cos m\pi \beta \cos n\pi \beta \end{array}$$

(A7) $$\begin{array}{l} v_{\beta \beta }+\frac{1}{2}\left(1+\nu \right)u_{\alpha \beta }+\frac{1}{2}\left(1-\nu \right)v_{\alpha \alpha }=\frac{1}{4}\pi^{3}\eta^{2}n\left(n^{2}-\nu m^{2}\right)\sin2n\pi \beta +\frac{1}{4}\pi^{3}\xi^{2}m\left(m^{2}-\nu n^{2}\right)\sin2m\pi \beta \\ -\frac{1}{4}\pi^{3}\eta^{2}n\left(m^{2}+n^{2}\right)\sin2n\pi \beta \cos2m\pi \alpha -\frac{1}{4}\pi^{3}\xi^{2}m\left(m^{2}+n^{2}\right)\sin2m\pi \beta \cos2n\pi \alpha \\ +\pi^{3}\eta \xi n\left[m^{2}+\frac{1}{2}\left(1-\nu \right)n^{2}\right]\sin m\pi \alpha \sin n\pi \alpha \sin m\pi \beta \cos n\pi \beta +\pi^{3}\eta \xi m\left[n^{2}+\frac{1}{2}\left(1-\nu \right)m^{2}\right]\sin m\pi \alpha \sin n\pi \alpha \cos m\pi \beta \sin n\pi \beta \\ -\frac{1}{2}\left(1+\nu \right)m^{2}n\pi^{3}\eta \xi \cos m\pi \alpha \cos n\pi \alpha \cos m\pi \beta \sin n\pi \beta -\frac{1}{2}\left(1+\nu \right)mn^{2}\pi^{3}\eta \xi \cos m\pi \alpha \cos n\pi \alpha \sin m\pi \beta \cos n\pi \beta \end{array}$$

After calculation, *u* and *v* can be derived from Equations (A6) and (A7) as follows:

(A8) $$\begin{array}{l} u=\frac{m\pi }{16}\eta^{2}(\cos2n\pi \beta -1+{\frac{\nu n}{m^{2}}}^{2})\sin2m\pi \alpha +\frac{n\pi }{16}\xi^{2}(\cos2m\pi \beta -1+{\frac{\nu m}{n^{2}}}^{2})\sin2n\pi \alpha \\ +\pi \eta \xi \left[a_{1}\sin m\pi \alpha \cos n\pi \alpha \sin m\pi \beta \sin n\pi \beta \right.+a_{2}\sin m\pi \alpha \cos n\pi \alpha \cos m\pi \beta \cos n\pi \beta \\ +a_{3}\cos m\pi \alpha \sin n\pi \alpha \sin m\pi \beta \sin n\pi \beta \left.+a_{4}\cos m\pi \alpha \sin n\pi \alpha \cos m\pi \beta \cos n\pi \beta \right] \end{array}$$

(A9) $$\begin{array}{l} v=\frac{n\pi }{16}\eta^{2}(\cos2m\pi \alpha -1+{\frac{\nu m}{n^{2}}}^{2})\sin2n\pi \beta +\frac{m\pi }{16}\xi^{2}(\cos2n\pi \alpha -1+{\frac{\nu n}{m^{2}}}^{2})\sin2m\pi \beta \\ +\pi \eta \xi \left[a_{1}\sin m\pi \alpha \sin n\pi \alpha \sin m\pi \beta \cos n\pi \beta \right.+a_{2}\cos m\pi \alpha \cos n\pi \alpha \sin m\pi \beta \cos n\pi \beta \\ +a_{3}\sin m\pi \alpha \sin n\pi \alpha \cos m\pi \beta \sin n\pi \beta \left.+a_{4}\cos m\pi \alpha \cos n\pi \alpha \cos m\pi \beta \sin n\pi \beta \right] \end{array}$$

The *a*_1_, *a*_2_, *a*_3_, and *a*_4_ are defined in the Appendix C. After substituting Equations (A5), (A8) and (A9) into Equation (13) and using the original symbols for *u* and *v*. The non-dimensional nonlinear plate motion equation can be expressed as follows:

(A10) $$\begin{array}{l} w_{\alpha \alpha \alpha \alpha }+2w_{\alpha \alpha \beta \beta }+w_{\beta \beta \beta \beta }=\tilde{F}-\gamma \ddot{w}-\zeta \dot{w}+\varepsilon \left\{12u_{\alpha }w_{\alpha \alpha }+6w_{\alpha }^{2}w_{\alpha \alpha }+12\nu \left(v_{\beta }w_{\alpha \alpha }+\frac{1}{2}w_{\beta }^{2}w_{\alpha \alpha }\right)\right.\\ +12v_{\beta }w_{\beta \beta }+6w_{\beta }^{2}w_{\beta \beta }+12\nu \left(u_{\alpha }w_{\beta \beta }+\frac{1}{2}w_{\alpha }^{2}w_{\beta \beta }\right)\left.+12(1-\nu )\left(u_{\beta }w_{\alpha \beta }+v_{\alpha }w_{\alpha \beta }+w_{\alpha }w_{\beta }w_{\alpha \beta }\right)\right\} \end{array}$$

## Appendix B

B. Nonlinear C-H-F-H plate equation

Consider a square clamped–hinged–free–hinged plate (C-H-F-H plate) with dimensions *a* and *b* where *a* = *b*. The displacement equations for *u*, *v* and *w* can be assumed to be $u=\bar{u}\phi_{n}(x)\phi_{m}(y)$, $v=\bar{v}\phi_{m}(y)\phi_{n}(x)$, $w=A(t)\phi_{n}(x)\phi_{m}(y)$, where A(t) represents the amplitude in the *w*-direction, and $\bar{u}$ and $\bar{v}$ are the vibration amplitudes coupled in the *x*-direction and *y*-direction of the plate. Here, $\phi_{n}(x)$ represents the vibration mode shape in the *x*-direction, with boundary conditions of clamped–free as shown in Equation (A11). Similarly, $\phi_{m}(y)$ represents the vibration mode shape in the *y*-direction, with Hinged-Hinged boundary conditions as shown in Equation (A12).

(A11) $$\phi_{n}(x)=\sin E_{n}x-\sinh E_{n}x-H_{n}(\cos E_{n}x-\cosh E_{n}x),\;H_{n}=\frac{\left(\sin E_{n}l+\sinh E_{n}l\right)}{\left(\cos E_{n}l+\cosh E_{n}l\right)}$$

(A12) $$\phi_{m}(y)=\sin(m\pi y)$$

Let α=x/a, β=y/b, τ=ωt, and continue using the original displacement notation *w*. Then, substitute $u=\bar{u}\phi_{n}(\alpha )\phi_{m}(\beta )$, $v=\bar{v}\phi_{m}(\beta )\phi_{n}(\alpha )$, into Equations (7) and (8) and follow the same steps as in the H-H-H-H plate, and after nondimensionalization, we obtain $\bar{u}$ and $\bar{v}$ as follows:

(A13) $$\bar{u}=-A{\left(t\right)}^{2}E_{n}\sin(m\pi \beta )(\cos E_{n}\alpha -\cosh E_{n}\alpha \;+H_{n}\sin E_{n}\alpha +H_{n}\sinh E_{n}\alpha )$$

(A14) $$\bar{v}=-A{\left(t\right)}^{2}(m\pi )\cos(m\pi \beta )(\sin E_{n}\alpha -\sinh E_{n}\alpha -H_{n}\cos E_{n}\alpha +H_{n}\cosh E_{n}\alpha )$$

Then, *u* and *v* can be expressed as follows:

(A15) $$u=-A{\left(t\right)}^{2}E_{n}\sin(m\pi \beta )(\cos E_{n}\alpha -\cosh E_{n}\alpha +H_{n}\sin E_{n}\alpha +H_{n}\sin E_{n}\alpha )\phi_{n}(\alpha )\phi_{m}(\beta )$$

(A16) $$v=-A{\left(t\right)}^{2}(m\pi )\cos(m\pi \beta )(\sin E_{n}\alpha -\sinh E_{n}\alpha -H_{n}\cos E_{n}\alpha +H_{n}\cosh E_{n}\alpha )\phi_{m}(\beta )\phi_{n}(\alpha )$$

Finally, by substituting Equations (A15) and (A16), and $w=A(t)\phi_{n}(\alpha )\phi_{m}(\beta )$ into the nonlinear plate motion Equation (13), and adding a damping term, where *μ* is the damping coefficient, we obtain the nonlinear plate motion equation with boundary conditions for a clamped–hinge–free–hinge plate. The resulting amplitude (*A*(*t*)) and spatial mode ($\phi_{n}(\alpha )\phi_{m}(\beta )$), after discretization, are as follows:

(A17) $$\ddot{A}Q_{1}(\alpha ,\beta )+\dot{A}Q_{2}(\alpha ,\beta )+AQ_{3}(\alpha ,\beta )-A^{3}Q_{4}(\alpha ,\beta )=\tilde{F}(t)$$

Here, *A* represents the amplitude in the *w*-direction, $\tilde{F}(t)$ represents the external force from the raindrops, and $Q_{1}(\alpha ,\beta )∼Q_{4}(\alpha ,\beta )$ are defined in Appendix C.

## Appendix C

$$\begin{array}{l} a_{1}=\frac{n\pi \left[m^{2}-n^{2}+\nu \left(m^{2}+n^{2}\right)\right]}{4\left(n^{2}-m^{2}\right)},\;a_{2}=\frac{mn^{2}\nu }{2\left(n^{2}-m^{2}\right)},\;a_{3}=\frac{m\pi \left[m^{2}-n^{2}-\nu \left(m^{2}+n^{2}\right)\right]}{4\left(n^{2}-m^{2}\right)},\;\;a_{4}=\frac{m^{2}n\nu }{2\left(m^{2}-n^{2}\right)}\\ Q_{1}(\alpha ,\beta )=\sin(m\pi \beta )(\sin E_{n}\alpha -\sinh E_{n}\alpha -H_{n}\cos E_{n}\alpha +H_{n}\cosh E_{n}\alpha )\\ Q_{2}(\alpha ,\beta )=\mu \sin(m\pi \beta )(\sin E_{n}\alpha -\sinh E_{n}\alpha -H_{n}\cos E_{n}\alpha +H_{n}\cosh E_{n}\alpha )\\ Q_{3}(\alpha ,\beta )={E_{n}}^{4}\sin(m\pi \beta )(\sin E_{n}\alpha -\sinh E_{n}\alpha -H_{n}\cos E_{n}\alpha +H_{n}\cosh E_{n}\alpha )\\ \;+{\left(m\pi \beta \right)}^{4}\sin(m\pi \beta )(\sin E_{n}\alpha -\sinh E_{n}\alpha -H_{n}\cos E_{n}\alpha +H_{n}\cosh E_{n}\alpha )\\ \;+2{E_{n}}^{2}{\left(m\pi \beta \right)}^{2}\sin(m\pi \beta )(\sin E_{n}\alpha +\sinh E_{n}\alpha -H_{n}\cos E_{n}\alpha -H_{n}\cosh E_{n}\alpha )\\ Q_{4}(\alpha ,\beta )=6{E_{n}}^{4}\sin{\left(m\pi \beta \right)}^{3}(\sin E_{n}\alpha +\sinh E_{n}\alpha -H_{n}\cos E_{n}\alpha \\ \;-H_{n}\cosh E_{n}\alpha ){\left(\cos E_{n}\alpha -\cosh E_{n}\alpha +H_{n}\sin E_{n}\alpha +H_{n}\sinh E_{n}\alpha \right)}^{2}\\ \;-6{\left(m\pi \beta \right)}^{4}\cos{\left(m\pi \beta \right)}^{2}\sin(m\pi \beta )(\sin E_{n}\alpha -\sinh E_{n}\alpha -H_{n}\cos E_{n}\alpha +H_{n}\cosh E_{n}\alpha {)}^{3}\\ \;-12{\left(m\pi \beta \right)}^{5}\cos(m\pi \beta )\sin{\left(m\pi \beta \right)}^{2}(\sin E_{n}\alpha -\sinh E_{n}\alpha \;-H_{n}\cos E_{n}\alpha +H_{n}\cosh E_{n}\alpha {)}^{3}\\ \;+6\nu {E_{n}}^{2}{\left(m\pi \beta \right)}^{2}\sin{\left(m\pi \beta \right)}^{3}(\cos E_{n}\alpha \\ \;-\cosh E_{n}\alpha +H_{n}\sin E_{n}\alpha +H_{n}\sinh E_{n}\alpha {)}^{2}(\sin E_{n}\alpha \\ \;-\sinh E_{n}\alpha \;-H_{n}\cos E_{n}\alpha +H_{n}\cosh E_{n}\alpha )\\ \;+{E_{n}}^{2}{\left(m\pi \beta \right)}^{2}\cos{\left(m\pi \beta \right)}^{2}\sin(m\pi \beta )(12\nu -12)(\cos E_{n}\alpha \\ \;-\cosh E_{n}\alpha +H_{n}\sin E_{n}\alpha +H_{n}\sinh E_{n}\alpha {)}^{2}(\sin E_{n}\alpha \\ \;\;-\sinh E_{n}\alpha -H_{n}\cos E_{n}\alpha +H_{n}\cosh E_{n}\alpha )\\ \;-6\nu {E_{n}}^{2}{\left(m\pi \beta \right)}^{2}\cos(m\pi \beta )\sin(m\pi \beta )(\cos(m\pi \beta )\\ \;+2(m\pi \beta )\sin(m\pi \beta ))(\sin E_{n}\alpha +\sinh E_{n}\alpha \\ \;-H_{n}\cos E_{n}\alpha -H_{n}\cosh E_{n}\alpha )(\sin E_{n}\alpha -\sinh E_{n}\alpha -H_{n}\cos E_{n}\alpha +H_{n}\cosh E_{n}\alpha {)}^{2} \end{array}$$
